# Effects of sodium benzoate on cognitive function in neuropsychiatric disorders: a systematic review and meta-analysis

**DOI:** 10.3389/fpsyt.2024.1370431

**Published:** 2024-09-09

**Authors:** Chun-Wei Liang, Hsiao-Yi Cheng, Mei-Chih Meg Tseng

**Affiliations:** ^1^ Department of Primary Care Medicine, Shuang Ho Hospital, Taipei Medical University, Taipei, Taiwan; ^2^ Department of Primary Care Medicine, Shin Kong Wu Ho-Su Memorial Hospital, Taipei, Taiwan; ^3^ Department of Psychiatry, Taipei Medical University Shuang Ho Hospital, New Taipei City, Taiwan; ^4^ Department of Psychiatry, School of Medicine, College of Medicine, Taipei Medical University, Taipei, Taiwan; ^5^ Department of Psychiatry, National Taiwan University College of Medicine, Taipei, Taiwan

**Keywords:** benzoate, cognitive function, dementia, schizophrenia, depression

## Abstract

We performed a systematic review and meta-analysis on sodium benzoate’s effects on cognitive function and other psychiatric symptoms in individuals with neuropsychiatric disorders. We searched PubMed, Embase, Cochrane Library, and PsychInfo databases until September 2023. A random-effects meta-analysis was performed within a frequentist framework. To investigate the potential sources of heterogeneity, we performed subgroup analyses based on sex, dose, diagnosis, and risk of bias of the included studies. Trial sequential analyses were performed to investigate the statistical power of the synthesized studies. The certainty in evidence was evaluated using the Grading of Recommendations Assessment, Development and Evaluation approach. A total of 10 studies were included in the analysis. Sodium benzoate demonstrated a small-to-moderate positive effect on global cognitive function compared with placebo (standardized mean difference 0.40, 95% confidence interval 0.20 to 0.60, high certainty). Subgroup analyses suggested more pronounced effects in women; individuals receiving doses >500 mg/day; and individuals with early-phase Alzheimer’s disease, chronic schizophrenia, or major depressive disorder. Sodium benzoate also demonstrated potential efficacy in enhancing the speed of processing, working memory, verbal learning and memory, visual learning and memory, and reasoning and problem solving. Furthermore, sodium benzoate was effective for positive psychotic symptoms but not for negative psychotic and depressive symptoms with moderate certainty. The current evidence strongly supports the positive effects of sodium benzoate on cognitive function in neuropsychiatric disorders. Further research is required to confirm its efficacy across different subtypes or stages of neurocognitive disorders and within specific cognitive domains.

**Systematic Review Registration:**

PROSPERO, identifier CRD42023457462

## 1 Introduction

The N-methyl-D-aspartate (NMDA) receptor, a subtype of ionotropic glutamate receptors, plays a critical role in processes such as learning, memory, and cognition (1, 2). Disruption of its signaling pathways has been implicated in the development of neuropsychiatric disorders, including Alzheimer’s disease, schizophrenia, and mood disorders (3). Studies have revealed diminished glutamate levels in both the cerebrospinal fluid and the brains of patients with Alzheimer’s disease (4, 5), along with a reduction in glutamate terminals within the hippocampus (6). Moreover, the serum levels of D-serine, an NMDA receptor agonist, were discovered to be lower in patients with Alzheimer’s disease (7). Preclinical investigations have demonstrated that ketamine, an NMDA receptor antagonist, enhanced positive, negative, and cognitive symptoms in not only healthy participants (8, 9) but also in patients with schizophrenia (10). Abnormal modulation of NMDA receptors, involving both enhancement and suppression of the NMDA receptor because of the presence of complex neural substrates, has also been implicated in depression (11). Studies have reported NMDA agonists (12–14) and antagonists (15, 16) to have potential antidepressant effects, highlighting the role of NMDA receptors in the pathophysiology of various neuropsychiatric disorders.

To address dysfunction of NMDA receptors, several NMDA-enhancing agents have been developed, showing potential positive effects on cognitive function in neuropsychiatric disorders (17, 18). Conventionally, activation of NMDA receptors involves the application of agonists such as D-serine (19) and D-cycloserine (20, 21). However, a novel approach to NMDA receptor activation has been proposed, which involves inhibiting the activity of D-amino acid oxidase (DAAO), a peroxisomal flavoenzyme responsible for degrading D-serine and D-alanine (22–24). The inhibition of DAAO activity leads to increased levels of D-amino acids, which activates the NMDA receptor through the coagonist site.

Sodium benzoate, as a DAAO inhibitor or an NMDA receptor modulator, may indirectly enhance the activity of NMDA receptors (25). Given the prevalence of NMDA hypofunction in various neuropsychiatric disorders, sodium benzoate was hypothesized to exert positive effects on cognitive function in these conditions, and a few clinical studies (26–37) on schizophrenia, neurocognitive disorders, and major depressive disorder have been conducted despite the precise mechanisms of sodium benzoate are yet to be fully understood (38). The efficacy of add-on sodium benzoate in improving cognitive function in patients with neuropsychiatric disorders was inconclusive. Several studies have presented promising results indicating improvement of global cognitive function after the use of add-on sodium benzoate in neurocognitive disorders (28, 30) and depressive disorder (36). Other studies have reported studies have reported unfavorable outcomes in various cognitive function domains (26, 27, 31–33). Two recent meta-analyses investigating the efficacy of add-on sodium benzoate on cognitive function in schizophrenia have revealed it does not lead to significant improvement (17, 39). Although considerable heterogeneity was present in one of the meta-analyses (39), no further analysis was conducted to explore the sources of the heterogeneity. The present systematic review and meta-analysis investigated the effects of sodium benzoate on the global and several specific domains of cognitive function. With the hypothesis of similar mechanisms of action of sodium benzoate in addressing NMDA hypofunction in various neuropsychiatric disorders, our meta-analysis encompassed a broad spectrum of conditions. Subgroup analyses were then undertaken to discern variances in sodium benzoate’s effects among different disorders, pinpointing potential factors influencing these effects. Additionally, we explored the effects of sodium benzoate on other neuropsychiatric symptoms such as positive and negative psychotic symptoms, depressive symptoms, and its safety profile.

## 2 Methods

### 2.1 Search strategy and selection criteria

This systematic review and meta-analysis was planned, conducted, and reported in accordance with the Preferred Reporting Items for Systematic Reviews and Meta-Analyses (PRISMA) guidelines (40). The protocol of this study was registered in PROSPERO (CRD42023457462).

We conducted a comprehensive search of PubMed, Embase, Cochrane Library, and PsycInfo databases (until September 2023) to identify randomized controlled trials (RCTs) investigating the efficacy and safety of sodium benzoate for neuropsychiatric disorders. The inclusion criteria for this study were determined on the basis of the Patient, Intervention, Comparison, Outcomes, and Study (PICOS) framework, and these criteria are summarized in **Supplementary Method S1**. The search strategy is outlined in **Supplementary Method S2**.

Two reviewers (CWL and HYC) independently gathered crucial data from the included studies. Any disagreement regarding trial inclusion or data extraction was resolved through consultation with a third reviewer (MCMT). The following data were extracted from each study: bibliography, grouping, benzoate dosage, diagnosis, patient age, illness onset age, years of education, body mass index (BMI), patient sex, sample size, intervention duration, and outcomes.

### 2.2 Risk-of-bias assessment

By using the revised Cochrane risk-of-bias tool for randomized trials (RoB 2; released on August 22, 2019), two reviewers (CWL and HYC) independently assessed the risk of bias of the included studies. Any disagreement was resolved through consultation with a third reviewer (MCMT).

### 2.3 Outcome measures

The primary outcomes in this study were those related to global cognitive function and seven cognitive domains, namely, speed of processing, sustained attention, working memory, verbal learning and memory, visual learning and memory, reasoning and problem solving, and social cognition. Global cognitive function was assessed using the MATRICS Consensus Cognitive Battery (MCCB) (26, 31, 32), the Alzheimer’s Disease Assessment Scale-Cognitive Subscale (ADAS-cog) (27, 28, 30, 33), and the Wechsler Adult Intelligence Scale (WAIS-III) (36) across the studies. Detailed information regarding the outcome measures used for assessing the seven cognitive domains across studies is presented in **Table 1**. The secondary outcomes were positive and negative psychotic symptoms, assessed using the Positive and Negative Symptoms of Schizophrenia Scale (PANSS), and depressive symptoms, assessed using the Hamilton Depression Rating Scale (HDRS). In terms of safety, the current study investigated all-cause dropout; all-cause adverse events; and extrapyramidal symptoms assessed using the Simpson–Angus Scale (SAS), the Abnormal Involuntary Movement Scale (AIMS), and the Barnes Akathisia Scale (BAS).

**Table 1:** Summary of findings.

Outcome	k	n	Outcome measure	Effect estimate (95%CI)	GRADE
Efficacy					
Global cognitive function	13	543	ADAS-cog, MCCB, and WAIS-III	0.40 (0.20 to 0.60)^a^	High
Speed of processing	11	434	Category Fluency, Trail Marking A, and WAIS-III digit symbol-coding	0.35 (0.14 to 0.56)^a^	High
Sustained attention	5	153	Continuous Performance Test	0.07 (−0.37 to 0.50)^a^	Low^d,e^
Working memory	11	432	Backward Digit Span, WAIS-III spatial span, and WAIS-III digit span	0.30 (0.10 to 0.51)^a^	High
Verbal learning and memory	11	436	WAIS-III word listing	0.33 (0.11 to 0.55)^a^	High
Visual learning and memory	5	152	WAIS-III visual reproduction	0.51 (0.17 to 0.85)^a^	Low^d,e^
Reasoning and problem solving	7	254	WAIS-III maze	0.39 (0.13 to 0.65)^a^	Moderate^d^
Social cognition	5	143	MSCEIT managing emotions branch	−0.08 (−0.42 to 0.24)^a^	Low^d,e^
Positive and negative symptoms	6	255	PANSS	−3.87 (−6.66 to −1.08)^b^	Moderate^d^
Positive symptoms	6	255	PANSS positive	−1.78 (−2.88 to −0.68)^b^	Moderate^d^
Negative symptoms	6	255	PANSS negative	−0.24 (−1.34 to 0.86)^b^	Moderate^d^
General psychopathology	6	255	PANSS general psychopathology	−1.16 (−2.65 to 0.33)^b^	Moderate^d^
Depressive symptoms	5	281	HDRS	-0.69 (-1.64 to 0.26)^b^	Moderate^d^
Safety					
All-cause dropout	12	679	Risk	0.79 (0.53 to 1.16)^c^	Moderate^e^
All-cause adverse event	11	658	Risk	0.98 (0.84 to 1.15)^c^	Moderate^e^
Extrapyramidal symptoms	4	156	SAS	0.34 (0.08 to 0.59)^b^	Low^d,e^
Dyskinesia	4	156	AIMS	−0.04 (−0.14 to 0.07)^b^	Moderate^d^
Akathisia	4	156	BAS	−0.00 (−0.03 to 0.02)^b^	Moderate^d^

^a^Standardized mean difference; ^b^Mean difference; ^c^Risk ratio; ^d^Reporting bias (potential asymmetry in the funnel plot); ^e^Imprecision (not crossing trial sequential monitoring boundaries, futility boundaries, or required information size in the trial sequential analysis).

ADAS-cog, The Alzheimer’s Disease Assessment Scale-Cognitive Subscale; AIMS, Abnormal Involuntary Movement Scale; BAS, Barnes Akathisia Scale; BEHAVE-AD, Behavioral Pathology in Alzheimer’s Disease Rating Scale; CI, confidence interval; GRADE, Grading of Recommendations Assessment, Development and Evaluation; HDRS, Hamilton Depression Rating Scale; k, comparison number; MCCB: the MATRICS Consensus Cognitive Battery; MSCEIT, Mayer-Salovey-Caruso Emotional Intelligence Test; n, patient number; PANSS, Positive and Negative Symptoms of Schizophrenia Scale; RR, risk ratio; SAS, Simpson-Angus Scale; WAIS-III, Wechsler Adult Intelligence Scale.

### 2.4 Data synthesis and analysis

A random-effects meta-analysis was performed using Review Manager (version 5.3). The data used for analysis comprised the change from baseline scores to postintervention scores. In cases in which studies did not report data for cognitive domains, composite scores were used for analysis. For continuous outcomes, the effect sizes are expressed as mean difference (MD) values when the same scales were used or as standardized mean difference (SMD) values when the effect estimates were pooled across studies using different scales, as occurred with global cognitive function and the seven cognitive domains investigated in this study. SMDs were calculated using Hedges’ g with a 95% confidence interval (CI). The interpretation of Hedges’ g aligns with that of Cohen’s d, with 0.2, 0.5, and 0.8 generally considered to indicate small, medium, and large effect sizes, respectively (41). For dichotomous outcomes, the effect sizes are presented as risk ratios (RRs). For studies with multiple treatment arms, each arm was considered a separate comparison group, with the overall group evenly split into two or more comparison groups. This approach allowed for approximate investigations of heterogeneity according to the Cochrane Handbook (42). The sample size for each treatment arm was calculated by dividing the original group’s size by the number of treatment arms.

To examine the global impact of sodium benzoate on cognitive function, this meta-analysis was conducted across various disorders. Additionally, subgroup analyses were performed to explore differences in the effects of sodium benzoate among different disorders and diagnoses. Further subgroup analyses were carried out to identify other potential effect modifiers and sources of heterogeneity, including sex (number or ratio), dosage, and risk of bias. Heterogeneity was assessed using the *I*² statistic, with *I*² > 50% indicating substantial heterogeneity. All subgroup differences were tested for significance, with *P* < 0.1 and *I*² > 50% indicating a significant subgroup effect (43).

To identify small study bias, funnel plots were constructed, and Egger regression asymmetry tests were performed (44). A *P* value of <0.1 indicated a significant small study bias.

We also conducted random-effects trial sequential analyses to investigate whether the total number of patients across the included studies provided sufficient statistical power for drawing definitive conclusions regarding the efficacy and safety of sodium benzoate in the treatment of neuropsychiatric disorders. The required information size was used to detect minimal clinically important differences (MCIDs) at 90% statistical power and a 5% type I error. Trial sequential analyses were performed using the Trial Sequential Analysis software (Copenhagen Trial Unit).

### 2.5 Certainty in evidence

The certainty in evidence was evaluated using the Grading of Recommendations Assessment, Development, and Evaluation (GRADE) approach, which categorizes evidence quality into four levels: very low, low, moderate, and high. These levels represent the degree of certainty of the effect estimates being consistent with the true effects (45). Five domains, namely risk of bias, reporting bias, indirectness, imprecision, and heterogeneity were assessed. To evaluate imprecision and heterogeneity, MCID values were calculated. Because our primary outcomes are presented in terms of SMDs, we back-transformed the SMDs to MCCB, ADAS-cog, and WAIS-III scores, assuming a standard deviation of 4.95, 5.97, and 4.70, respectively. The MCID for global cognitive function was considered to be 3 points on the ADAS-cog (46), corresponding to an SMD of 0.5. The MCID for positive and negative symptoms was set at 5 points on the PANSS (37), that for depressive symptoms was set at 4 points on the HDRS (47), that for all-cause dropout and all-cause adverse events was set at 25% risk reduction or increase, and that for extrapyramidal symptoms was set at 0.3 points on the SAS (48).

## 3 Results

### 3.1 Characteristics and risk of bias of the included studies

**Figure 1:**
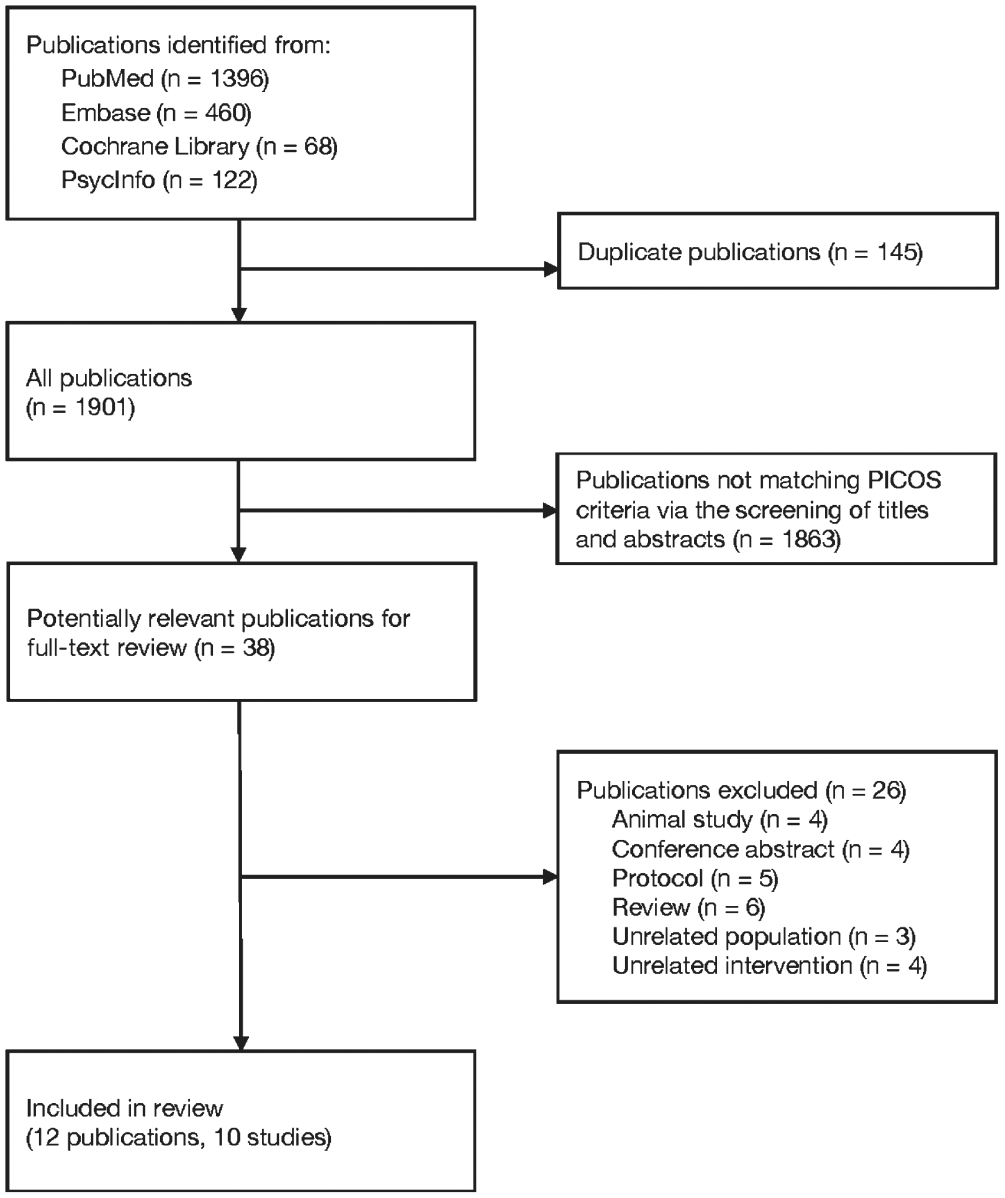
Flowchart depicting study selection process.

**Figure 1** illustrates the study selection process. The initial search of the databases yielded 1,901 publications. After the titles and abstracts were screened to eliminate publications that did not align with the PICOS criteria for study inclusion (**Supplementary Method S1**), 38 publications were selected for full-text review. Subsequently, 26 articles were excluded (**Supplementary Table S1**), resulting in identification of 12 publications (26–37), comprising 10 RCTs (**Supplementary Table S2**).

These RCTs, published between 2013 and 2023, comprised four studies involving schizophrenia or psychotic disorders (26, 31, 32, 37); five focusing on neurocognitive disorders, such as Alzheimer’s disease, dementia with behavioral and psychological symptoms (BPSD), and mild cognitive impairment (27–30, 33–35); and one centered on major depressive disorder (37). All studies assessed the efficacy of add-on sodium benzoate with acetylcholine esterase inhibitor/antipsychotics, with the exception of one that analyzed the efficacy of sodium benzoate alone in patients with major depressive disorder (37). The dose of sodium benzoate ranged from 250 to 2000 mg/day, with some studies using fixed doses (26, 28, 31, 32, 37) and the others using flexibly adjusted doses based on patient response and tolerability (27, 29, 30, 33–36). The intervention duration ranged from 6 to 24 weeks, and the sample size ranged from 9 to 50 patients per treatment arm.

The results of the risk-of-bias assessment are outlined in **Supplementary Table S3**. A low risk of bias was observed, with bias arising from the randomization process in 8 trials, deviations from intended interventions in 7, missing outcome data in 7, measurement of the outcome in 10, and selection of the reported result in 5. Regarding the overall risk of bias, half of the studies had a low risk of bias (27, 30, 32, 37), and the other half had some concerns of risk of bias (26, 28, 31, 33–36).

### 3.2 Primary outcome: cognitive function

**Figure 2:**
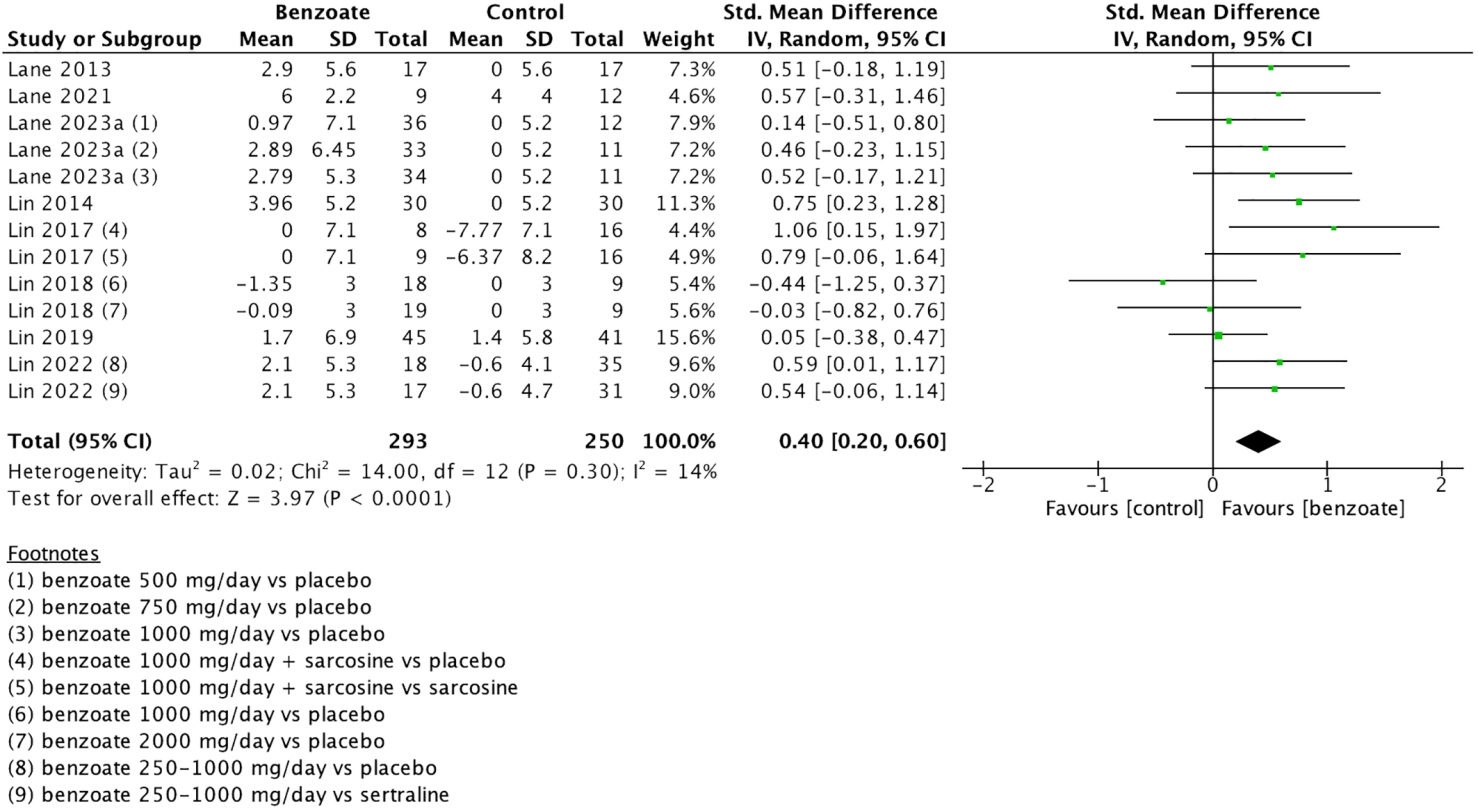
Forest plot of global cognitive function. Each point estimate (square) denotes the comparison effect (standardized mean difference) of the outcome, and the horizontal lines represent the 95% confidence intervals. Results to the right of the vertical line indicate effects favoring benzoate. The black diamond represents the combined effect. Abbreviations: CI, confidence interval; df, degrees of freedom; IV, inverse variance; SD, standard deviation; std, standardized.

Nine of the 10 included studies assessed cognitive function. Of them, one employed concurrent transcranial direct current stimulation (tDCS) as an adjunct to add-on sodium benzoate (29), and it was excluded from the main analysis due to concerns that the addition of neuromodulation treatments might have influenced its findings regarding the efficacy of sodium benzoate (29). The main analysis was based on the 8 studies encompassing 13 comparisons and involving 543 patients. Sodium benzoate exerted a small-to-moderate positive effect on global cognitive function compared with placebo (SMD 0.40, 95% CI 0.20 to 0.60, *P <*0.0001; **Table 1**; **Figure 2**). The meta-analysis exhibited low heterogeneity (*I*² = 14%; **Figure 2**). We further investigated the effects of sodium benzoate on seven cognitive domains (**Supplementary Figure S1**) and discovered that sodium benzoate exerted small-to-moderate positive effects on most cognitive function domains, including speed of processing (SMD 0.35, 95% CI, 0.14 to 0.56), working memory (SMD 0.30, 95% CI, 0.10 to 0.51), verbal learning and memory (SMD 0.33, 95% CI, 0.11 to 0.55), visual learning and memory (SMD 0.51, 95% CI, 0.17 to 0.85), and reasoning and problem solving (SMD 0.39, 95% CI, 0.13 to 0.65) with low heterogeneity (*I*² ranging from 0% to 14%). However, sodium benzoate may exert no effect on sustained attention or social cognition.

**Figure 3:**
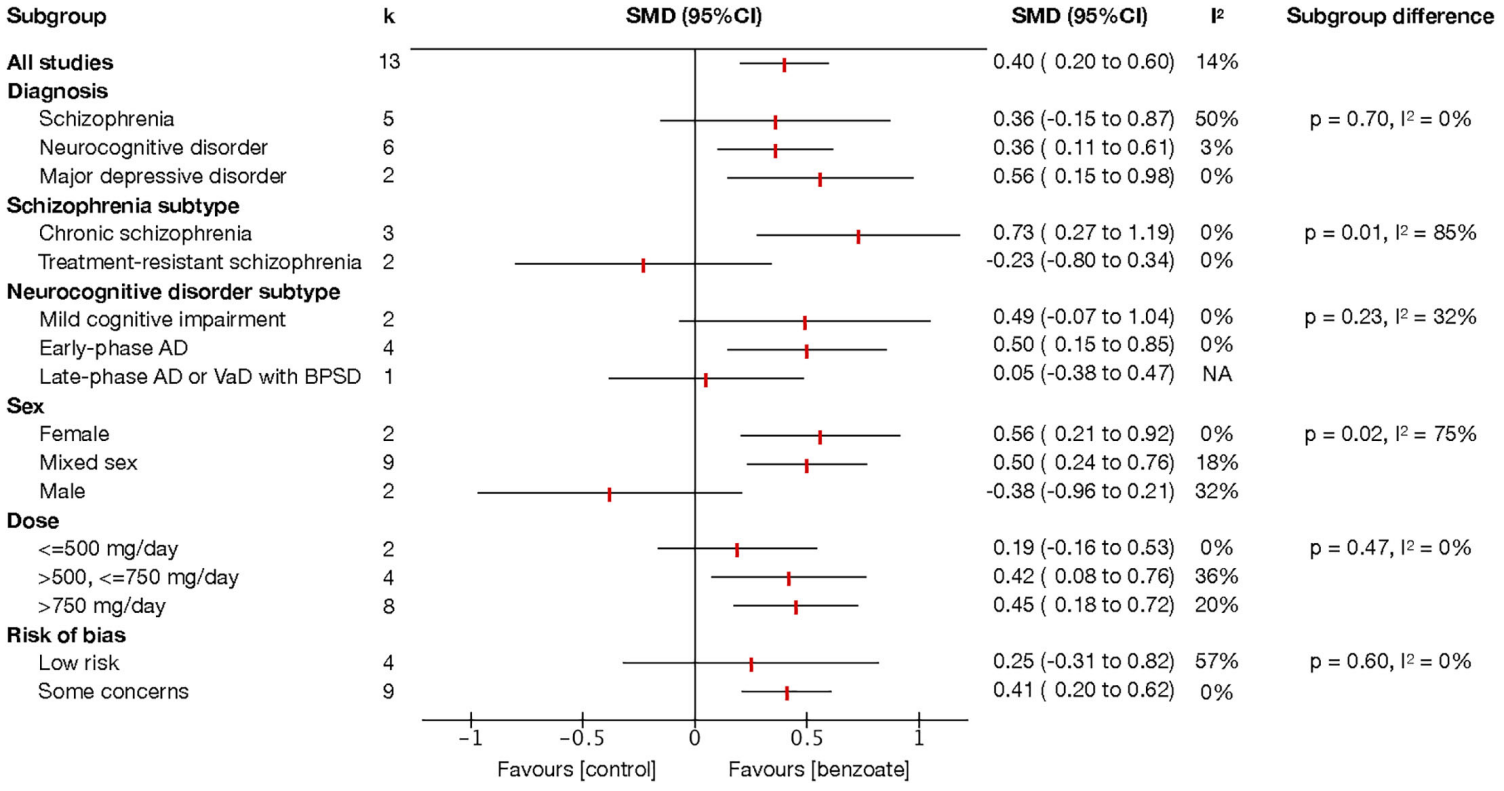
Subgroup analysis of global cognitive function. Each point estimate represents the comparison effect (standardized mean difference) of the outcome in the subgroup, and the horizontal lines represent the 95% confidence intervals. Results to the right of the vertical line indicate effects favoring benzoate. Abbreviations: AD, Alzheimer’s disease; BPSD, behavioral and psychological symptoms of dementia; CI, confidence interval; SMD, standardized mean difference; VaD, vascular dementia.

Subgroup analyses of global cognitive function revealed significant differences in the sex (*P* = 0.02, *I*² = 75%) and schizophrenia subtype subgroups (*P* = 0.01, *I*² = 85%) (**Figure 3**). Sodium benzoate exerted a significantly greater effect on global cognitive function in women (SMD 0.56, 95% CI, 0.21 to 0.92) than in men (SMD −0.38, 95% CI, −0.96 to 0.21). The effect of add-on sodium benzoate on global cognitive function significantly differed between the patients with chronic schizophrenia and those with treatment-resistant schizophrenia.

Although no significant subgroup differences were identified among diagnostic categories, sodium benzoate was demonstrated to be significantly beneficial for patients with neurocognitive disorders (SMD 0.36, 95% CI, 0.11 to 0.61) and major depressive disorder (SMD 0.56, 95% CI, 0.15 to 0.98) when its effects were compared with those of placebo, with low heterogeneity (**Figure 3**). Sodium benzoate exhibited no significant effects on global cognitive function compared with placebo in patients with schizophrenia, although considerable heterogeneity was noted in this analysis. A subgroup analysis of the studies involving schizophrenia revealed that add-on sodium benzoate had significant beneficial effects on global cognitive function in chronic schizophrenia (SMD 0.73, 95% CI, 0.27 to 1.19), but not in treatment-resistant schizophrenia when compared with placebo. For neurocognitive disorders, add-on sodium benzoate appeared to have more positive benefits with a moderate effect size in patients with early-phase Alzheimer’s disease (SMD 0.50, 95% CI, 0.15 to 0.85) when compared with placebo than in patients with mild cognitive impairment and dementia with BPSD. Moreover, sodium benzoate had efficacy in improving global cognitive function in patients receiving doses exceeding 500 mg/day (SMDs for >500, ≤750 mg/day and >750 mg/day were 0.42 [95% CI, 0.08 to 0.76], and 0.45 [95% CI, 0.18 to 0.72], respectively).

Subgroup analyses were performed for the seven cognitive domains (**Supplementary Table S4**). The majority of the findings in the subgroup analyses of the five domains that favored sodium benzoate aligned with the results of the subgroup analyses of global cognitive function, with a few exceptions. Sodium benzoate may not exhibit significantly greater efficacy than placebo in improving the domains of speed of processing, working memory, and verbal learning and memory in early Alzheimer’s disease. However, add-on sodium benzoate may demonstrate greater efficacy in improving the domains of speed of processing, verbal learning and memory, and visual learning and memory in patients with chronic schizophrenia.

To further validate the results of our analysis, we conducted a subgroup analysis based on risk of bias (**Figure 3**). Additionally, we performed sensitivity analyses in which the study with concurrent tDCS (29) was included and the two treatment arms using an active control (31, 36) were excluded (**Supplementary Table S5**). The subgroup difference in the analysis based on risk of bias was nonsignificant (*P* = 0.60, *I*² = 0%). The results of the sensitivity analysis (which included the study with concurrent tDCS [SMD 0.33, 95% CI, 0.10 to 0.56, *P* = 0.006] and excluded the two treatment arms with an active control [SMD 0.35, 95% CI, 0.14 to 0.57, *P* = 0.001]) were consistent with those of the main analysis. However, the inclusion of the study with concurrent tDCS (29) substantially increased the heterogeneity (*I*² = 46%), justifying the exclusion of the study from the main analysis.

### 3.3 Secondary outcome: positive and negative psychotic symptoms

According to an analysis of 4 studies involving 6 comparisons and 255 patients (**Table 1**; **Supplementary Figure S2**), add-on sodium benzoate significantly reduced the PANSS total score (MD −3.87, 95% CI, −6.66 to −1.08, *P* = 0.007) and PANSS positive score (MD −1.78, 95% CI, −2.88 to −0.68, *P* = 0.002). However, add-on sodium benzoate had no significant effect on the PANSS negative score or PANSS general psychopathology score.

The results of the subgroup analyses of different PANSS subscales are presented in **Supplementary Table S6**. Significant sex differences were noted for both the PANSS total score (*P* = 0.08, *I*² = 68%) and PANSS positive score (*P* = 0.08, *I*² = 67%). Studies with a higher female ratio revealed significantly greater improvements in positive and total symptoms following the use of add-on sodium benzoate (MD −1.66, 95% CI, −2.89 to −0.43). Although no significant difference was noted for any PANSS subscale in the diagnosis subgroup analysis, the results of the analysis indicated that patients with early psychosis (MD −0.30, 95% CI, −1.95 to 1.35) may benefit the least from add-on sodium benzoate in terms of positive symptoms.

### 3.4 Secondary outcome: depressive symptoms

According to an analysis of 4 studies involving 5 comparisons and 281 patients (**Table 1**; **Supplementary Figure S3**), sodium benzoate did not exert significant effects on HDRS (MD −0.69, 95% CI, −1.64 to 0.26, *P* = 0.15). However, our subgroup analyses (data not shown) revealed that female patients may experience a greater reduction in depressive symptoms than male patients do.

### 3.5 Safety

Sodium benzoate was generally well tolerated (**Table 1**; **Supplementary Figure S4**) and did not lead to higher risks of dropping out (RR 0.80, 95% CI, 0.54 to 1.18) or adverse events (RR 0.98, 95% CI, 0.84 to 1.15). However, sodium benzoate may be associated with a slight increase in extrapyramidal symptoms in patients with schizophrenia (MD 0.34, 95% CI, 0.08 to 0.59, *P* = 0.009).

### 3.6 Publication bias

Funnel plots were constructed, as presented in **Supplementary Figure S5**. Regarding the global cognitive function outcome, the Egger regression asymmetry test indicated nonsignificant publication bias (*P* = 0.50). For the other outcomes, because the number of studies investigating the outcomes was limited (≤10 comparisons), we could not perform Egger regression asymmetry tests.

### 3.7 Trial sequential analysis

**Figure 4:**
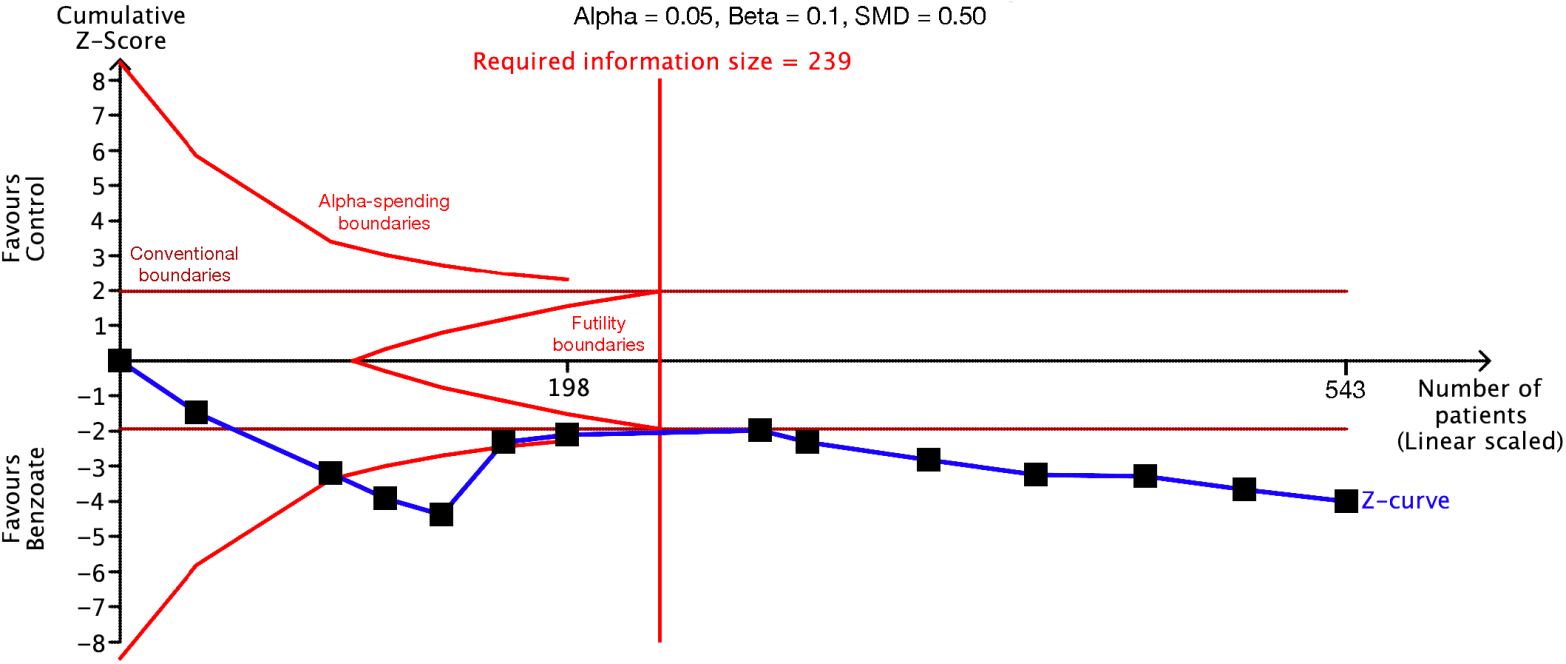
Trial sequential analysis of global cognitive function. The required information size was used to detect a standardized mean difference of 0.50 with 90% statistical power and a 5% type I error. The sample size exceeded the required information size, indicating that the evidence supporting the benefit of benzoate is unlikely to be overturned by future randomized controlled trials.

The trial sequential analysis demonstrated that the sample sizes for the studies investigating global cognitive function and the cognitive domains of speed of processing, working memory, verbal learning and memory, and reasoning and problem solving exceeded the information sizes required to achieve sufficient statistical power (**Figure 4**; **Table 1**; **Supplementary Figure S6**). However, in the analysis of the cognitive domains of sustained attention, visual learning and memory, and social cognition, the number of patients did not exceed the required information size. The Z-curves also did not cross the alpha-spending boundaries or the futility boundaries (**Table 1**; **Supplementary Figure S6**). Consequently, current evidence is insufficient for definitive conclusions to be drawn on the basis of an analysis of these cognitive domains. Regarding secondary outcomes and the safety profile, the analyses of positive and negative symptoms and depressive symptoms had sufficient statistical power (**Table 1**; **Supplementary Figure S7**). However, the statistical power of the analyses of all-cause dropout, all-cause adverse events, and extrapyramidal symptoms may be insufficient (data not shown).

### 3.8 Certainty in evidence

We observed high certainty of evidence for the effect of sodium benzoate on global cognitive function and the domains of speed of processing, working memory, and verbal learning and memory (**Table 1**); moderate certainty of evidence for the results for the reasoning and problem-solving domain; and low certainty of evidence for the results across the sustained attention, visual learning and memory, and social cognition domains. Additionally, we observed moderate certainty of evidence for the results for positive and negative symptoms, depressive symptoms, all-cause dropout, and all-cause adverse events and low certainty of evidence for the increase in extrapyramidal symptoms following sodium benzoate treatment.

## 4 Discussion

This is the first systematic review and meta-analysis on sodium benzoate’s effects on various neuropsychiatric disorders. Synthesis of the current evidence yielded sufficient statistical power to confirm the benefits of sodium benzoate for global cognitive function and domains of speed of processing, working memory, and verbal learning and memory. Moreover, sodium benzoate demonstrated potential effectiveness in enhancing visual learning and memory as well as reasoning and problem solving. However, no significant effects were identified on sustained attention and social cognition. Subgroup analyses revealed sodium benzoate may exhibit greater efficacy in improving global cognitive function in women, patients receiving sodium benzoate doses >500 mg/day, patients with neurocognitive disorders (especially early-phase Alzheimer’s disease), chronic schizophrenia, and major depressive disorder. Our findings indicate that sodium benzoate may be beneficial for addressing positive symptoms of schizophrenia but may have no significant effects on negative symptoms and depressive symptoms. Regarding safety, sodium benzoate is generally well tolerated but is potentially associated with slightly increased extrapyramidal symptoms in patients with schizophrenia.

Compared with the findings of prior studies (27, 28, 30, 33), those of the present meta-analysis enable us to report with increased confidence on the positive effect of sodium benzoate on global cognitive function in patients with neurocognitive disorders. Notably, our meta-analysis indicates that sodium benzoate may be more efficacious in patients with early stage Alzheimer’s disease than in those with mild cognitive impairment or dementia with BPSD. Moreover, our study findings reveal that sodium benzoate had positive effects on several domains of cognitive function that have only been reported on in few previous studies (26, 30). With its large sample size, this meta-analysis has a higher level of confidence in affirming the positive effects of sodium benzoate on domains such as speed of processing, working memory, and verbal learning and memory than previous studies have had. However, given that available data are limited, additional clinical studies are required to validate the efficacy of sodium benzoate across various subtypes and stages of neurocognitive disorders and within specific cognitive domains.

Prior to our study, two meta-analyses have investigated the effects of sodium benzoate on cognitive function in patients with schizophrenia (17, 39). In line with the meta-analysis conducted by Chang et al. (17) in 2019 and that by Seetharam et al. (39) in 2022, our study revealed that the addition of benzoate did not result in significant improvements in cognitive function in patients with schizophrenia compared with placebo. Nevertheless, the subgroup analysis of our study revealed that add-on sodium benzoate might be beneficial for improving global cognitive function in individuals with chronic schizophrenia. Moreover, our subgroup analysis of cognitive domains indicated positive effects of add-on sodium benzoate on the domains of speed of processing, verbal learning and memory, and visual learning and memory in patients with chronic schizophrenia. Our results suggest that add-on sodium benzoate is a potential cognitive enhancer useful for patients with schizophrenia who are not treatment-resistant to antipsychotics. For patients with treatment-resistant schizophrenia, they often had severer symptoms and longer duration of illness and were commonly prescribed with clozapine. Prior studies have reported that pharmacotherapy augmentations of clozapine showed some effects on cognitive function in treatment-resistant schizophrenia (49) but the findings were not firmly established (50). To our knowledge, patients with treatment-resistant schizophrenia also failed to show improvement in cognitive function in most of the previous trials with NMDA-enhancing agents, including D-serine (51), glycine (52, 53), and D-cycloserine (54). Whether sodium benzoate has poorer effects on treatment-resistant schizophrenia alone or has a negative synergic effect with clozapine on cognitive function among these patients warrants further investigations.

In our subgroup analyses, sex emerged as a crucial effect modifier for the influence of benzoate treatment across the dimensions of cognitive function, psychotic symptoms, and depressive symptoms. Women who receive such treatment may experience greater improvements in cognitive function, positive symptoms of schizophrenia, and depressive symptoms than men do. The observed sex difference in the response to sodium benzoate was hypothesized to be linked to the potential role of benzoate in mimicking the action of sexual hormones (35). In *in vivo* and *in vitro* studies, derivatives of benzoate, including p-hydroxybenzoate and various alkyl hydroxy benzoate preservatives, exhibited estrogenic effects (55, 56). These sexual hormones and their mimetics may subsequently modulate the expression of hippocampal NMDA receptors and interact with circulating antioxidants (57, 58), thereby affecting the cognitive function in patients with neuropsychiatric disorders. However, in Lin et al. (35), the levels of neither estradiol nor FSH were altered by benzoate. Although a significant increase in the estradiol-to-FSH ratio was observed in patients receiving sodium benzoate, the effect size may be too small to be clinically relevant. Larger studies with longer follow-up durations are warranted to elucidate the mechanisms underlying the sex difference in the treatment effect of sodium benzoate.

In the subgroup analyses of our study, significant cognitive benefits of sodium benzoate were observed in patients receiving doses greater than 500 mg/day. Prior to our study, no consensus had been achieved regarding the optimal dose of benzoate; a wide range of benzoate dosages were used in the studies included in our analysis, with these dosages ranging from 250 to 2,000 mg/day. A dose-finding trial conducted in 2023 (28) reported that the cognitive benefits of sodium benzoate reached statistical significance in groups being administered doses of 750 or 1,000 mg/day, whereas no significant effect was identified in the group being administered 500 mg/day. The subgroup analysis in our study further confirmed the efficacy of sodium benzoate in improving global cognitive function in patients receiving doses exceeding 500 mg/day.

In the meta-analysis by Seetharam et al. (39), sodium benzoate demonstrated potential benefits with respect to improving positive psychotic symptoms but exhibited no effects on negative symptoms, general psychopathology, or the total PANSS score. Regarding safety, the administration of sodium benzoate did not appear to increase the risk of adverse events. Our results were mostly consistent with theirs, with a notable exception. In our study, sodium benzoate demonstrated efficacy in reducing the total PANSS score. Hypotheses regarding the role of NMDA receptors in schizophrenia have been raised. The efficacy of NMDA receptors determines the integrity of synaptic spines within the neural networks. Failure of the ErbB4 receptors is responsible for NMDA receptor decline, with concomitant regression of synaptic spines resulting in changes in neural network function that underlie the positive and negative symptoms of schizophrenia (59, 60) According to our findings, we postulate that negative symptoms might be less involved in the above hypothetical mechanism than positive symptoms was.

In this study, we observed a mild increase in SAS scores following sodium benzoate treatment. Neurotransmitter alterations, including the hypoactivity of dopamine and gamma-aminobutyric acid (GABA) and the hyperactivity of acetylcholine and glutamate were involved in antipsychotics-induced Parkinsonism (61). Memantine, an NMDA antagonist, may alleviate extrapyramidal symptoms by reducing glutamate hyperactivity and inhibiting presynaptically D2 dopaminergic neurons in the putamen (62). On the other hand, NMDA-enhancing agents, including sodium benzoate, may therefore increase the severity of extrapyramidal symptoms. Cautions should be exercised in using sodium benzoate, particularly for patients with schizophrenia.

In addition to inhibition of DAAO activity, sodium benzoate may demonstrate potential neuroprotective effects by mitigating oxidative stress and related inflammatory processes, based on *in vitro* and *in vivo* evidence (63–65). In prior clinical research (28, 32), sodium benzoate has shown an ability to elevate levels of catalase and glutathione, both vital endogenous antioxidants implicated in the pathophysiology of various neuropsychiatric disorders. Since the exact mechanisms of sodium benzoate are still elusive, exploring its antioxidative and anti-inflammatory properties could expand our comprehension of its precise mode of action.

In this study, we performed trial sequential analyses in addition to traditional meta-analyses. The trial sequential analysis method was derived from that of interim analysis, which is used in clinical trials, and it establishes more stringent boundaries of significance to mitigate the risk of inflation in both type I and II errors stemming from multiple and sequential testing (66). In cases where the number of included studies was inadequate and the statistical power of the meta-analysis was insufficient, a Z score higher than the traditional level of significance of 1.96σ was required for a difference to be considered significant (67). In this study, the Z-curve for global cognitive function surpassed both the alpha-spending boundary for benefit and the required information size. This indicates that the evidence supporting the efficacy of sodium benzoate for global cognitive function is robust and unlikely to be overturned by future studies.

This study has some limitations. First, incorporating studies across various neuropsychiatric disorders into the meta-analysis was based on the hypothesis of the prevalence of NMDA hypofunction in cognitive impairment in these conditions and comparable mechanisms of action of sodium benzoate. This methodology has also been adopted by previous meta-analyses to evaluate the efficacy of treatments across a wide range of psychiatric disorders (68, 69). This approach not only enhances the statistical power of the meta-analysis but also allows for subgroup analyses to investigate whether the treatment’s efficacy is consistent across different disorders. However, the validity of this hypothesis may vary, particularly given the incomplete understanding of mechanisms of sodium benzoate as suggested by previous laboratory research (38). The subgroup analyses might also be underpowered and susceptible to confounding and ecological biases. Hence, future studies should focus on elucidating the precise mechanisms of sodium benzoate across a spectrum of neuropsychiatric disorders. Second, the majority of the included studies was conducted by the same team in Taiwan, which may limit the generalizability of the results. In our study, low-to-moderate heterogeneity was observed in the analysis of positive psychotic symptoms, and it mainly stemmed from the study by Scott et al. (37) conducted in Australia. Whether sodium benzoate has different effects among patients with diverse ethnic backgrounds needs further research to examine. Third, a few potential outcome predictors other than sex, such as lower baseline behavioral and psychotic symptoms of dementia (34), higher BMI (34), younger age (34), and higher baseline catalase levels (28), have been reported to be associated with greater improvement in ADAS-cog in some of the included studies. However, due to the unavailability of individual participant data, we were unable to perform analyses of these predictors in this study. Fourth, most of the studies included in our analysis assessed the efficacy of add-on sodium benzoate rather than benzoate alone. Whether add-on benzoate treatments (e.g., sarcosine, acetylcholine esterase inhibitors, or antipsychotics) would have synergic effects different from those of benzoate alone remains unknown. Additional studies as well as individual participant data meta-analyses are warranted in the future to address some of the aforementioned limitations.

In conclusion, our meta-analysis yielded sufficient statistical power and achieved high certainty in evidence, enabling us to affirm the positive impact of sodium benzoate on global cognitive function. The effects of sodium benzoate appear to be more pronounced in women; individuals receiving benzoate doses higher than 500 mg/day; and those with chronic schizophrenia, early-phase Alzheimer’s disease, or major depressive disorders. Additionally, sodium benzoate may be effective in enhancing speed of processing, working memory, verbal learning and memory, visual learning and memory, and reasoning and problem-solving. However, no significant effects were observed on sustained attention and social cognition. For patients with schizophrenia, benzoate should be prescribed with caution because it may increase the risk of extrapyramidal symptoms occurring.

## Data Availability

The original contributions presented in the study are included in the article/**Supplementary Material**. Further inquiries can be directed to the corresponding author.
