# Supplementary material for: Effects of sodium benzoate on cognitive function in neuropsychiatric disorders: a systematic review and meta-analysis
Source: Front Psychiatry. 2024 Sep 9;15:1370431. doi: 10.3389/fpsyt.2024.1370431 (PMC11416944; doi:10.3389/fpsyt.2024.1370431)
Supplement: Supplementary file 1 [file DataSheet1.docx]

**SUPPLEMETARY INFORMATION**

**Method S1. Eligibility criteria**

**Method S2. Search strategy**

**Table S1. Excluded studies**

**Table S2. Included studies**

**Table S3. Risk of bias assessment**

**Table S4. Subgroup analyses of seven cognitive domains**

**Table S5. Sensitivity analyses of global cognitive function**

**Table S6. Subgroup analyses of the positive and negative symptoms**

**Figure S1. Forest plots of seven cognitive domains**

**Figure S2. Forest plots of the positive and negative symptoms**

**Figure S3. Forest plot of depressive symptoms**

**Figure S4. Forest plots of the safety outcome**

**Figure S5. Funnel plots of publication bias**

**Figure S6. Trial sequential analyses of seven cognitive domains**

**Figure S7. Trial sequential analyses of secondary outcomes**

**Method S1. Eligibility criteria**

PICOS

Patient: neuropsychiatric disorders

Intervention: benzoate

Comparison: placebo, or drugs that have been shown to exert similar effects to placebo

Outcome: cognitive function, positive and negative symptoms of schizophrenia, depression, all-cause dropout, all-cause adverse event, and extrapyramidal symptoms

Study: randomized controlled trials

**Method S2. Search strategy**

PubMed:

benzoate AND (psychiatric disorder OR neuropsychiatric disorder OR major neurocognitive disorder OR dementia OR Alzheimer OR Alzheimer’s disease OR mild cognitive impairment OR schizophrenia OR psychosis OR depression OR anxiety) AND (randomized OR randomization OR controlled trial OR clinical trial OR placebo)

Embase:

('mental disease'/exp OR 'abnormal mental state' OR 'disease, mental' OR 'diseased mental state' OR 'disorder, mental' OR 'disordered mental state' OR 'disturbed mental state' OR 'illness, mental' OR 'insanity' OR 'mental abnormality' OR 'mental change' OR 'mental confusion' OR 'mental defect' OR 'mental disease' OR 'mental disorder' OR 'mental disorders' OR 'mental disorders diagnosed in childhood' OR 'mental disturbance' OR 'mental illness' OR 'mental insufficiency' OR 'mental symptom' OR 'mentally ill' OR 'neurodevelopmental disorder' OR 'neurodevelopmental disorders' OR 'neuropsychiatric disease' OR 'neuropsychiatric diseases' OR 'neuropsychiatric disorder' OR 'neuropsychiatric disorders' OR 'psychiatric disease' OR 'psychiatric disorder' OR 'psychiatric illness' OR 'psychiatric symptom' OR 'psychic disease' OR 'psychic disorder' OR 'psychic disturbance' OR 'psychologic disorder' OR 'psychologic disturbance' OR 'psychological disorder' OR 'psychological disturbance' OR 'psychopathology' OR 'major neurocognitive disorder'/exp OR 'dementia'/exp OR 'amentia' OR 'dementia' OR 'demention' OR 'alzheimer disease'/exp OR 'alzeimer disease' OR 'alzeimer`s disease' OR 'alzeimers disease' OR 'alzheimer dementia' OR 'alzheimer disease' OR 'alzheimers disease' OR 'alzheimer fibrillary change' OR 'alzheimer fibrillary lesion' OR 'alzheimer neurofibrillary change' OR 'alzheimer neurofibrillary degeneration' OR 'alzheimer neuron degeneration' OR 'alzheimer perusini disease' OR 'alzheimer sclerosis' OR 'alzheimer syndrome' OR 'alzheimer`s disease' OR 'cortical sclerosis, diffuse' OR 'dementia, alzheimer' OR 'diffuse cortical sclerosis' OR 'late onset alzheimer disease' OR 'mild cognitive impairment'/exp OR 'amnestic mild cognitive impairment' OR 'mild cognitive impairment' OR 'schizophrenia'/exp OR 'childhood schizophrenia' OR 'chronic schizophrenia' OR 'dementia praecox' OR 'dementia precox' OR 'schizophrenia' OR 'schizophrenia, childhood' OR 'schizophrenic' OR 'schizophrenic language' OR 'schizophrenic syndrome' OR 'psychosis'/exp OR 'atypical psychosis' OR 'cycloid psychosis' OR 'encephalopschychosis' OR 'first episode psychosis' OR 'presenile psychosis' OR 'psychoses, substance-induced' OR 'psychosis' OR 'psychosis, cycloid' OR 'psychosis, toxic' OR 'psychosis, unipolar' OR 'psychotic' OR 'psychotic condition' OR 'psychotic disorder' OR 'psychotic disorders' OR 'psychotic episode' OR 'psychotic experience' OR 'psychotic patient' OR 'psychotic period' OR 'psychotic reaction' OR 'psychotic states' OR 'psychotic symptom' OR 'psychoticism' OR 'reaction psychosis' OR 'schizophrenia and disorders with psychotic features' OR 'schizophrenia spectrum and other psychotic disorders' OR 'substance-induced psychoses' OR 'symbiotic psychosis' OR 'toxic psychosis' OR 'unipolar psychosis' OR 'depression'/exp OR 'central depression' OR 'clinical depression' OR 'depression' OR 'depressive disease' OR 'depressive disorder' OR 'depressive episode' OR 'depressive illness' OR 'depressive personality disorder' OR 'depressive state' OR 'depressive symptom' OR 'depressive syndrome' OR 'mental depression' OR 'parental depression' OR 'anxiety'/exp OR 'anxiety') AND benzoate AND ('randomization'/exp OR 'random allocation' OR 'randomisation' OR 'randomization' OR 'controlled study'/exp OR 'control group study' OR 'control group trial' OR 'controlled study' OR 'controlled trial' OR 'randomized controlled trial'/exp OR 'controlled trial, randomized' OR 'randomised controlled study' OR 'randomised controlled trial' OR 'randomized controlled study' OR 'randomized controlled trial' OR 'trial, randomized controlled' OR 'clinical trial'/exp OR 'clinical drug trial' OR 'clinical trial' OR 'major clinical trial' OR 'trial, clinical')

Cochrane Library:

#1 benzoate

#2 psychiatric disorder OR neuropsychiatric disorder OR major neurocognitive disorder OR dementia OR Alzheimer OR Alzheimer’s disease OR mild cognitive impairment OR schizophrenia OR psychosis OR depression OR anxiety

#3 randomized OR randomization OR controlled trial OR clinical trial OR placebo

#4 #1 AND #2 AND #3

PsycInfo:

benzoate AND (psychiatric disorder OR neuropsychiatric disorder OR major neurocognitive disorder OR dementia OR Alzheimer OR Alzheimer’s disease OR mild cognitive impairment OR schizophrenia OR psychosis OR depression OR anxiety)

**Table S1. Excluded studies**

| **Reason for exclusion** | **Title** | **First Author** | **Publication Year** |
| --- | --- | --- | --- |
| Animal study | A novel NMDA receptor-based intervention to suppress compulsion-like alcohol drinking | Wegner SA | 2019 |
| Animal study | D-amino acid oxidase is expressed in the ventral tegmental area and modulates cortical dopamine | Betts JF | 2014 |
| Animal study | DREAM/calsenilin/KChIP3 modulates strategy selection and estradiol-dependent learning and memory | Tunur T | 2013 |
| Animal study | Novel mechanism of regulation of N-methyl-D-aspartate neurotransmission for CNS disorders | Tsai G | 2012 |
| Conference abstract | P521. Preliminary Results From a Multi-Centre, Double-Blind, Randomised Placebo Controlled Feasibility Trial of Add-on Sodium Benzoate and/or N-acetylcysteine in Patients With Early Schizophrenia | Husain M | 2022 |
| Conference abstract | Sodium benzoate as an adjunctive treatment in early psychosis: A randomised clinical trial | Scott JG | 2021 |
| Conference abstract | A randomized, double-blind, placebo-controlled add-on treatment of benzoate, a d-amino acid oxidase inhibitor, for schizophrenia | Tsai GE | 2012 |
| Conference abstract | Benzoate, a d-amino acid oxidase inhibitor, for treatment of early-phase alzheimer's disease: A randomized, double-blind, placebo-controlled trial | Tsai GE | 2013 |
| Protocol | Sodium benzoate for treatment of attenuated psychosis | EUCTR2013-000458-23-FI | 2013 |
| Protocol | The efficacy of sodium benzoate as an adjunctive treatment in early psychosis - CADENCE-BZ: study protocol for a randomized controlled trial | Ryan A | 2017 |
| Protocol | Sodium Benzoate and/or N-Acetylcysteine Added to TAU in Patients With Early Schizophrenia Spectrum Disorder | NCT03510741 | 2018 |
| Protocol | Dose finding clinical trial of sodium benzoate in people with treatment refractory schizophrenia | ACTRN12621000327886 | 2021 |
| Protocol | Protocol update and statistical analysis plan for CADENCE-BZ: a randomized clinical trial to assess the efficacy of sodium benzoate as an adjunctive treatment in early psychosis | Lim C | 2019 |
| Review | Augmentation Strategies for Clozapine-Resistant Patients with Schizophrenia | Chiu YH | 2020 |
| Review | Clozapine combined with different antipsychotic drugs for treatment-resistant schizophrenia | Barber S | 2017 |
| Review | Efficacy and safety of add-on sodium benzoate, a D-amino acid oxidase inhibitor, in treatment of schizophrenia: A systematic review and meta-analysis | Seetharam JC | 2022 |
| Review | Novel treatment for the most resistant schizophrenia: Dual activation of NMDA receptor and antioxidant | Lin CH | 2020 |
| Review | Treatment of NMDA agonist, glycine transporter i Inhibitor and D-Amino acid oxidase inhibitor for mental disorders | Lane HY | 2012 |
| Review | Zotepine versus other atypical antipsychotics for schizophrenia | Subramanian S | 2010 |
| Unrelated intervention | Antipsychotic combination using low-dose antipsychotics is as efficacious and safe as, but cheaper, than optimal-dose monotherapy in the treatment of schizophrenia: a randomized, double-blind study | Lin CH | 2013 |
| Unrelated intervention | Deanol in the treatment of hyperkinetic children | Coleman N | 1976 |
| Unrelated intervention | Dose equivalents of antidepressants: Evidence-based recommendations from randomized controlled trials | Hayasaka Y | 2015 |
| Unrelated intervention | Retention rates in placebo- and nonplacebo-controlled clinical trials of schizophrenia | Labelle A | 1999 |
| Unrelated population | Pharmacokinetics and Safety of Sodium Benzoate, a d-Amino Acid Oxidase (DAAO) Inhibitor, in Healthy Subjects: A Phase I, Open-label Study | Lin YS | 2022 |
| Unrelated population | The effects of a double blind, placebo controlled, artificial food colourings and benzoate preservative challenge on hyperactivity in a general population of preschool children | Leickly FE | 2005 |
| Unrelated population | The effects of a double blind, placebo controlled, artificial food colourings and benzoate preservative challenge on hyperactivity in a general population sample of preschool children | Bateman B | 2004 |

**Table S2. Included studies**

| **Study** | **Group** | **Diagnosis** | **Criteria for diagnosis** | **Age (yr)** | **Illness onset age (yr)** | **Education (yr)** | **BMI (kg/m^2^)** | **Female ratio** | **N** | **Intervention duration** |
| --- | --- | --- | --- | --- | --- | --- | --- | --- | --- | --- |
| Lane 2013^1^ | Benzoate 1000 mg/day | Schizophrenia | DSM-IV | 38.4 | 22.2 | 11.1 | BW 68.6 | 0.56 | 25 | 6 wk |
|  | Placebo |  |  | 36.3 | 23.4 | 10.5 | BW 64.4 | 0.44 | 27 |  |
| Lane 2021^2^ | Benzoate 250-1500 mg/day | MCI (CDR .5) | NINCDS-ADRDA | 66.1 | 65.4 | 5 | 22.7 | 0.78 | 9 | 24 wk |
|  | Placebo |  |  | 69.2 | 68.9 | 8.8 | 24 | 0.41 | 12 |  |
| Lane 2023a^3^ | Benzoate 500 mg/day | AD (CDR 1) | NINCDS-ADRDA | 75.2 | 74 | 5.2 | 25.1 | 0.61 | 38 | 24 wk |
|  | Benzoate 750 mg/day |  |  | 73.8 | 72.7 | 5.9 | 24.7 | 0.69 | 36 |  |
|  | Benzoate 1000 mg/day |  |  | 74.2 | 73.7 | 5.2 | 24.1 | 0.68 | 37 |  |
|  | Placebo |  |  | 75.8 | 74.1 | 5.3 | 23.2 | 0.61 | 38 |  |
| Lane 2023b^4^ | Benzoate 250-1500 mg/day + tDCS | AD (CDR 1) or MCI (CDR .5) | NINCDS-ADRDA | 73.7 | 71.5 | 6.2 | 23.5 | 0.70 | 47 | 24 wk |
|  | Placebo + tDCS |  |  | 75.0 | 71.8 | 6.5 | 24.8 | 0.60 | 50 |  |
| Lin 2014^5^ | Benzoate 250-750 mg/day | AD (CDR 1) or MCI (CDR .5) | NINCDS-ADRDA | 70.7 | 69.8 | 5.9 | 24.6 | 0.6 | 30 | 24 wk |
|  | Placebo |  |  | 69.7 | 68.5 | 7.5 | 23.9 | 0.63 | 30 |  |
| Lin 2017^6^ | Benzoate 1000 mg/day + Sarcosine | Schizophrenia | DSM-IV | 37.8 | 25.4 | 11.1 | 21.9 | 0.48 | 21 | 12 wk |
|  | Sarcosine |  |  | 38.2 | 23.7 | 11.7 | 21.5 | 0.29 | 21 |  |
|  | Placebo |  |  | 39.1 | 24.1 | 11.6 | 21.9 | 0.31 | 21 |  |
| Lin 2018^7^ | Benzoate 1000 mg/day | Treatment-resistant schizophrenia | DSM-IV | 44.3 | 22.5 | 10.5 | 23.1 | 0.30 | 20 | 6 wk |
|  | Benzoate 2000 mg/day |  |  | 44.8 | 23.6 | 11.0 | 23.7 | 0.35 | 20 |  |
|  | Placebo |  |  | 47 | 21.2 | 10.4 | 24.4 | 0.30 | 20 |  |
| Lin 2019^8^, 2020^9^, 2021^10^ | Benzoate 250-1500 mg/day | AD or VaD (CDR 1-3) with BPSD | NINCDS-ADRDA | 75.7 | 74.1 | 4.6 | 22.7 | 0.61 | 49 | 6 wk |
|  | Placebo |  |  | 75.2 | 72.7 | 4.8 | 24.2 | 0.67 | 48 |  |
| Lin 2022^11^ | Benzoate 250-1500 mg/day | Major depressive disorder | DSM-IV | 66.4 | NA | 7.5 | 22.9 | 0.69 | 39 | 8 wk |
|  | Sertraline |  |  | 66.9 | NA | 6.7 | 23.7 | 0.87 | 39 |  |
|  | Placebo |  |  | 70.5 | NA | 6.7 | 24.2 | 0.74 | 39 |  |
| Scott 2020^12^ | Benzoate 1000 mg/day | Psychotic disorder | DSM-IV | 21.7 | Disease duration <2 yr | NA | 25.8 | 0.32 | 49 | 12 wk |
|  | Placebo |  |  | 21.2 | Disease duration <2 yr | NA | 27.7 | 0.30 | 50 |  |

Abbreviations: AD, Alzheimer’s disease; BPSD, behavioral and psychological symptoms of dementia; CDR, Clinical Dementia Rating Scale; DSM, Diagnostic and Statistical Manual of Mental Disorders; MCI, mild cognitive impairment; NINCDS-ADRDA, National Institute of Neurological and Communicative Disorders and Stroke and the Alzheimer’s Disease and Related Disorders Association; tDCS, transcranial direct current stimulation; VaD, vascular dementia; wk, week; yr, year.

References:

1. Lane H-Y, Lin C-H, Green MF, Hellemann G, Huang C-C, Chen P-W, et al. Add-on treatment of benzoate for schizophrenia: a randomized, double-blind, placebo-controlled trial of D-amino acid oxidase inhibitor. JAMA Psychiatry. 2013; 70: 1267–75.

2. Lane H-Y, Tu C-H, Lin W-C, Lin C-H. Brain Activity of Benzoate, a D-Amino Acid Oxidase Inhibitor, in Patients with Mild Cognitive Impairment in a Randomized, Double-Blind, Placebo Controlled Clinical Trial. Int. J. Neuropsychopharmacol. 2021; 24: 392–9.

3. Lane H-Y, Wang S-H, Lin C-H. Endogenous antioxidants predicted outcome and increased after treatment: A benzoate dose-finding, randomized, double-blind, placebo-controlled trial for Alzheimer’s disease. Psychiatry Clin. Neurosci. 2023; 77: 102–9.

4. Lane H-Y, Wang S-H, Lin C-H. Adjunctive transcranial direct current stimulation (tDCS) plus sodium benzoate for the treatment of early-phase Alzheimer’s disease: A randomized, double-blind, placebo-controlled trial. Psychiatry Res. 2023; 328: 115461.

5. Lin C-H, Chen P-K, Chang Y-C, Chuo L-J, Chen Y-S, Tsai GE, et al. Benzoate, a D-Amino Acid Oxidase Inhibitor, for the Treatment of Early-Phase Alzheimer Disease: A Randomized, Double-Blind, Placebo-Controlled Trial. Biol. Psychiatry. 2014; 75: 678–85.

6. Lin C-Y, Liang S-Y, Chang Y-C, Ting S-Y, Kao C-L, Wu Y-H, et al. Adjunctive sarcosine plus benzoate improved cognitive function in chronic schizophrenia patients with constant clinical symptoms: A randomised, double-blind, placebo-controlled trial. World J. Biol. Psychiatry. 2017; 18: 357–68.

7. Lin C-H, Lin C-H, Chang Y-C, Huang Y-J, Chen P-W, Yang H-T, et al. Sodium Benzoate, a D-Amino Acid Oxidase Inhibitor, Added to Clozapine for the Treatment of Schizophrenia: A Randomized, Double-Blind, Placebo-Controlled Trial. Biol. Psychiatry. 2018; 84: 422–32.

8. Lin C-H, Chen P-K, Wang S-H, Lane H-Y. Sodium benzoate for the treatment of behavioral and psychological symptoms of dementia (BPSD): A randomized, double-blind, placebo-controlled, 6-week trial. J. Psychopharmacol. 2019; 33: 1030–3.

9. Lin C-H, Yang H-T, Chen P-K, Wang S-H, Lane H-Y. Precision Medicine of Sodium Benzoate for the Treatment of Behavioral and Psychological Symptoms of Dementia (BPSD). Neuropsychiatr. Dis. Treat. 2020; 16: 509–18.

10. Lin C-H, Chen P-K, Wang S-H, Lane H-Y. Effect of Sodium Benzoate on Cognitive Function Among Patients With Behavioral and Psychological Symptoms of Dementia: Secondary Analysis of a Randomized Clinical Trial. JAMA Netw Open. 2021; 4: e216156–e216156.

11. Lin C-H, Wang S-H, Lane H-Y. Effects of Sodium Benzoate, a D-Amino Acid Oxidase Inhibitor, on Perceived Stress and Cognitive Function Among Patients With Late-Life Depression: A Randomized, Double-Blind, Sertraline- and Placebo-Controlled Trial. Int. J. Neuropsychopharmacol. 2022; 25: 545–55.

12. Scott JG, Baker A, Lim CCW, Foley S, Dark F, Gordon A, et al. Effect of Sodium Benzoate vs Placebo Among Individuals With Early Psychosis: A Randomized Clinical Trial. JAMA Netw Open. 2020; 3: e2024335.

**Table S3. Risk of bias assessment**

| **Study** | **Bias arising from the randomization process** | **Bias due to deviations from intended interventions** | **Bias due to missing outcome data** | **Bias in measurement of the outcome** | **Bias in selection of the reported result** | **Overall risk of bias** |
| --- | --- | --- | --- | --- | --- | --- |
| Lane 2013 | Some concerns | Low risk | Low risk | Low risk | Low risk | Some concerns |
| Lane 2021 | Low risk | Low risk | Low risk | Low risk | Low risk | Low risk |
| Lane 2023a | Low risk | Some concerns | Some concerns | Low risk | Low risk | Some concerns |
| Lane 2023b | Low risk | Low risk | Low risk | Low risk | Low risk | Low risk |
| Lin 2014 | Low risk | Low risk | Low risk | Low risk | Low risk | Low risk |
| Lin 2017 | Low risk | Low risk | Low risk | Low risk | Some concerns | Some concerns |
| Lin 2018 | Low risk | Low risk | Low risk | Low risk | Low risk | Low risk |
| Lin 2019, 2020, 2021 | Some concerns | Some concerns | Some concerns | Low risk | Low risk | Some concerns |
| Lin 2022 | Low risk | Some concerns | Some concerns | Low risk | Low risk | Some concerns |
| Scott 2020 | Low risk | Low risk | Low risk | Low risk | Low risk | Low risk |

**Table S4. Subgroup analyses of seven cognitive domains**

| **Subgroup** | **k** | **SMD (95%CI)** | **I^2^** | **Subgroup difference** |
| --- | --- | --- | --- | --- |
| 1. **Speed of processing** | | | | |
| All studies | 11 | 0.35 (0.14 to 0.56) | 6% | NA |
| **Diagnosis** | | | | |
| Schizophrenia | 5 | 0.25 (-0.16 to 0.66) | 30% | *P* = 0.51, I^2^ = 0% |
| Neurocognitive disorder | 4 | 0.30 (-0.07 to 0.66) | 19% |  |
| Major depressive disorder | 3 | 0.56 (0.15 to 0.98) | 0% |  |
| **Schizophrenia subtype** | | | | |
| Chronic schizophrenia | 3 | 0.56 (0.14 to 0.98) | 0% | *P* = 0.02, I^2^ = 80% |
| Treatment-resistant schizophrenia | 2 | -0.25 (-0.82 to 0.31) | 0% |  |
| **Neurocognitive disorder subtype** | | | | |
| Mild cognitive disorder | 1 | 0.68 (-0.07 to 1.44) | NA | *P* = 0.28, I^2^ = 13% |
| Early-phase AD | 4 | 0.23 (-0.13 to 0.59) | 0% |  |
| **Sex** | | | | |
| Female ratio ≥50% | 7 | 0.44 (0.21 to 0.68) | 0% | *P* = 0.16, I^2^ = 49% |
| Female ratio <50% | 4 | 0.09 (-0.34 to 0.52) | 10% |  |
| **Dose** | | | | |
| ≤500 mg/day | 1 | 0.13 (-0.35 to 0.60) | NA | *P* = 0.28, I^2^ = 22% |
| >500, ≤750 mg/day | 2 | 0.60 (0.24 to 0.97) | 0% |  |
| >750 mg/day | 8 | 0.35 (0.10 to 0.60) | 5% |  |
| 1. **Sustained attention** | | | | |
| All studies | 5 | 0.07 (-0.37 to 0.50) | 38% | NA |
| **Schizophrenia subtype** | | | | |
| Chronic schizophrenia | 3 | 0.37 (-0.04 to 0.78) | 0% | *P* = 0.02, I^2^ = 82% |
| Treatment-resistant schizophrenia | 2 | -0.47 (-1.05 to 0.10) | 0% |  |
| **Sex** | | | | |
| Female ratio ≥50% | 1 | 0.27 (-0.29 to 0.84) | NA | *P* = 0.49, I^2^ = 0% |
| Female ratio <50% | 4 | -0.01 (-0.59 to 0.57) | 48% |  |
| 1. **Working memory** | | | | |
| All studies | 11 | 0.30 (0.10 to 0.51) | 0% | NA |
| **Diagnosis** | | | | |
| Schizophrenia | 5 | 0.13 (-0.20 to 0.47) | 0% | *P* = 0.29, I^2^ = 20% |
| Neurocognitive disorder | 4 | 0.30 (-0.07 to 0.66) | 19% |  |
| Major depressive disorder | 3 | 0.56 (0.15 to 0.98) | 0% |  |
| **Schizophrenia subtype** | | | | |
| Chronic schizophrenia | 3 | 0.25 (-0.17 to 0.67) | 0% | *P* = 0.36, I^2^ = 0% |
| Treatment-resistant schizophrenia | 2 | -0.08 (-0.64 to 0.48) | 0% |  |
| **Neurocognitive disorder subtype** | | | | |
| Mild cognitive disorder | 1 | 0.68 (-0.07 to 1.44) | NA | *P* = 0.28, I^2^ = 13% |
| Early-phase AD | 4 | 0.23 (-0.13 to 0.59) | 0% |  |
| **Sex** | | | | |
| Female ratio ≥50% | 7 | 0.34 (0.11 to 0.58) | 0% | *P* = 0.50, I^2^ = 0% |
| Female ratio <50% | 4 | 0.18 (-0.23 to 0.59) | 0% |  |
| **Dose** | | | | |
| ≤500 mg/day | 1 | 0.13 (-0.35 to 0.60) | NA | *P* = 0.23, I^2^ = 33% |
| >500, ≤750 mg/day | 2 | 0.60 (0.24 to 0.97) | 0% |  |
| >750 mg/day | 8 | 0.28 (0.03 to 0.52) | 0% |  |
| 1. **Verbal learning and memory** | | | | |
| All studies | 11 | 0.33 (0.11 to 0.55) | 14% | NA |
| **Diagnosis** | | | | |
| Schizophrenia | 5 | 0.20 (-0.22 to 0.63) | 35% | *P* = 0.46, I^2^ = 0% |
| Neurocognitive disorder | 4 | 0.30 (-0.07 to 0.66) | 19% |  |
| Major depressive disorder | 3 | 0.56 (0.15 to 0.98) | 0% |  |
| **Schizophrenia subtype** | | | | |
| Chronic schizophrenia | 3 | 0.50 (0.09 to 0.91) | 0% | *P* = 0.02, I^2^ = 81% |
| Treatment-resistant schizophrenia | 2 | -0.32 (-0.89 to 0.24) | 0% |  |
| **Neurocognitive disorder subtype** | | | | |
| Mild cognitive disorder | 1 | 0.68 (-0.07 to 1.44) | NA | *P* = 0.28, I^2^ = 13% |
| Early-phase AD | 4 | 0.23 (-0.13 to 0.59) | 0% |  |
| **Sex** | | | | |
| Female ratio ≥50% | 7 | 0.40 (0.17 to 0.64) | 0% | *P* = 0.39, I^2^ = 0% |
| Female ratio <50% | 4 | 0.13 (-0.44 to 0.70) | 46% |  |
| **Dose** | | | | |
| ≤500 mg/day | 1 | 0.13 (-0.35 to 0.60) | NA | *P* = 0.26, I^2^ = 26% |
| >500, ≤750 mg/day | 2 | 0.60 (0.24 to 0.97) | 0% |  |
| >750 mg/day | 8 | 0.31 (0.05 to 0.58) | 15% |  |
| 1. **Visual learning and memory** | | | | |
| All studies | 5 | 0.51 (0.17 to 0.85) | 0% | NA |
| **Schizophrenia subtype** | | | | |
| Chronic schizophrenia | 3 | 0.61 (0.19 to 1.03) | 0% | *P* = 0.42, I^2^ = 0% |
| Treatment-resistant schizophrenia | 2 | 0.32 (-0.24 to 0.89) | 0% |  |
| **Sex** | | | | |
| Female ratio ≥50% | 1 | 0.70 (0.11 to 1.28) | NA | *P* = 0.44, I^2^ = 0% |
| Female ratio <50% | 4 | 0.42 (0.01 to 0.83) | 0% |  |
| 1. **Reasoning and problem solving** | | | | |
| All studies | 7 | 0.39 (0.13 to 0.65) | 0% | NA |
| **Diagnosis** | | | | |
| Schizophrenia | 5 | 0.27 (-0.06 to 0.61) | 0% | *P* = 0.29, I^2^ = 13% |
| Major depressive disorder | 2 | 0.56 (0.15 to 0.98) | 0% |  |
| **Schizophrenia subtype** | | | | |
| Chronic schizophrenia | 3 | 0.25 (-0.20 to 0.70) | 0% | *P* = 0.81, I^2^ = 0% |
| Treatment-resistant schizophrenia | 2 | 0.34 (-0.23 to 0.91) | 0% |  |
| **Sex** | | | | |
| Female ratio ≥50% | 3 | 0.41 (0.07 to 0.74) | 0% | *P* = 0.85, I^2^ = 0% |
| Female ratio <50% | 4 | 0.35 (-0.06 to 0.77) | 0% |  |
| 1. **Social cognition** | | | | |
| All studies | 5 | -0.08 (-0.42 to 0.27) | 0% | NA |
| **Schizophrenia subtype** | | | | |
| Chronic schizophrenia | 3 | 0.04 (-0.3 to 0.47) | 0% | *P* = 0.39, I^2^ = 0% |
| Treatment-resistant schizophrenia | 2 | -0.28 (-0.84 to 0.29) | 0% |  |
| **Sex** | | | | |
| Female ratio ≥50% | 1 | -0.01 (-0.64 to 0.62) | NA | *P* = 0.80, I^2^ = 0% |
| Female ratio <50% | 4 | -0.11 (-0.51 to 0.30) | 0% |  |

Because none of the studies reported the treatment effect of benzoate in different sexes, we could only perform the subgroup analysis by female ratio.

Abbreviations: AD, Alzheimer’s disease; CI, confidence interval; k, study number; MD, mean difference.

**Table S5. Sensitivity analyses of global cognitive function**

|  | **k** | **SMD (95%CI)** | ***P* value** | ***I*^2^** |
| --- | --- | --- | --- | --- |
| Main analysis | 13 | 0.40 (0.20 to 0.60) | <0.0001 | 14% |
| Including the study with concurrent tDCS | 14 | 0.33 (0.10 to 0.56) | 0.006 | 46% |
| Excluding treatment arms with active control | 11 | 0.35 (0.14 to 0.57) | 0.001 | 13% |

Treatment arms with active control: benzoate 1000 mg/day + sarcosine vs placebo in Lin 2017 and benzoate 250-1000 mg/day vs sertraline in Lin 2022.

Abbreviations: CI, confidence interval; k, study number; SMD, standardized mean difference, tDCS, transcranial direct current stimulation.

**Table S6. Subgroup analyses of the positive and negative symptoms outcome**

| **Subgroup** | **k** | **MD (95%CI)** | **I^2^** | **Subgroup difference** |
| --- | --- | --- | --- | --- |
| 1. **PANSS total** | | | | |
| All studies | 6 | -3.87 (-6.66 to -1.08) | 0% | NA |
| **Diagnosis** | | | | |
| Early psychosis | 1 | -1.20 (-6.05 to 3.65) | NA | *P* = 0.45, I^2^ = 0% |
| Chronic schizophrenia | 3 | -4.79 (-10.28 to 0.69) | 31% |  |
| Treatment-resistant schizophrenia | 2 | -5.44 (-10.65 to -0.24) | 0% |  |
| **Sex** | | | | |
| Female ration ≥50% | 1 | -9.70 (-16.73 to -2.67) | NA | *P* = 0.08, I^2^ = 68% |
| Female ration <50% | 5 | -2.78 (-5.82 to -0.26) | 0% |  |
| **Risk of bias** | | | | |
| Low risk | 3 | -4.79 (-10.28 to 0.69) | 31% | *P* = 0.63, I^2^ = 0% |
| Some concerns/high risk | 3 | -3.17 (-6.72 to 0.38) | 0% |  |
| 1. **PANSS positive** | | | | |
| All studies | 6 | -1.78 (-2.88 to -0.68) | 38% | NA |
| **Diagnosis** | | | | |
| Early psychosis | 1 | -0.30 (-1.95 to 1.35) | NA | *P* = 0.11, I^2^ = 54% |
| Chronic schizophrenia | 3 | -1.86 (-3.51 to -0.20) | 44% |  |
| Treatment-resistant schizophrenia | 2 | -2.81 (-4.53 to -1.10) | 0% |  |
| **Sex** | | | | |
| Female ration ≥50% | 1 | -3.50 (-5.66 to -1.34) | NA | *P* = 0.08, I^2^ = 67% |
| Female ration <50% | 5 | -1.39 (-2.42 to -0.35) | 16% |  |
| **Risk of bias** | | | | |
| Low risk | 3 | -1.86 (-3.51 to -0.20) | 44% | *P* = 0.95, I^2^ = 0% |
| Some concerns/high risk | 3 | -1.78 (-3.60 to 0.04) | 54% |  |
| 1. **PANSS negative** | | | | |
| All studies | 6 | -0.24 (-1.34 to 0.86) | 0% | NA |
| **Diagnosis** | | | | |
| Early psychosis | 1 | 0.10 (-3.27 to 3.47) | NA | *P* = 0.71, I^2^ = 0% |
| Chronic schizophrenia | 3 | -1.08 (-2.93 to 0.78) | 0% |  |
| Treatment-resistant schizophrenia | 2 | -1.59 (-3.82 to 0.64) | 0% |  |
| **Sex** | | | | |
| Female ration ≥50% | 1 | -2.30 (-5.12 to 0.52) | NA | *P* = 0.12, I^2^ = 59% |
| Female ration <50% | 5 | 0.14 (-1.06 to 1.33) | 0% |  |
| **Risk of bias** | | | | |
| Low risk | 3 | -1.08 (-2.93 to 0.78) | 0% | *P* = 0.27, I^2^ = 17% |
| Some concerns/high risk | 3 | 0.22 (-1.15 to 1.59) | 0% |  |
| 1. **PANSS general psychopathology** | | | | |
| All studies | 6 | -1.16 (-2.65 to 0.33) | 0% | NA |
| **Diagnosis** | | | | |
| Early psychosis | 1 | 0.60 (-2.18 to 3.38) | NA | *P* = 0.34, I^2^ = 8% |
| Chronic schizophrenia | 3 | -1.92 (-4.32 to 0.47) | 0% |  |
| Treatment-resistant schizophrenia | 2 | -1.82 (-4.45 to 0.81) | 0% |  |
| **Sex** | | | | |
| Female ration ≥50% | 1 | -3.70 (-7.40 to 0.00) | NA | *P* = 0.14, I^2^ = 54% |
| Female ration <50% | 5 | -0.67 (-2.30 to 0.96) | 0% |  |
| **Risk of bias** | | | | |
| Low risk | 3 | -1.92 (-4.32 to 0.47) | 0% | *P* = 0.42, I^2^ = 0% |
| Some concerns/high risk | 3 | -0.68 (-2.58 to 1.23) | 0% |  |

Because none of the studies with PANSS outcomes reported the treatment effect of benzoate in different sexes, we could only perform the subgroup analysis by female ratio.

We did not perform subgroup analyses by dose because all of the studies used benzoate >750 mg/day.

Abbreviations: CI, confidence interval; k, study number; MD, mean difference; PANSS, Positive and Negative Symptoms of Schizophrenia Scale.

**Figure S1. Forest plots of seven cognitive domains**

1. **Speed of processing**

**
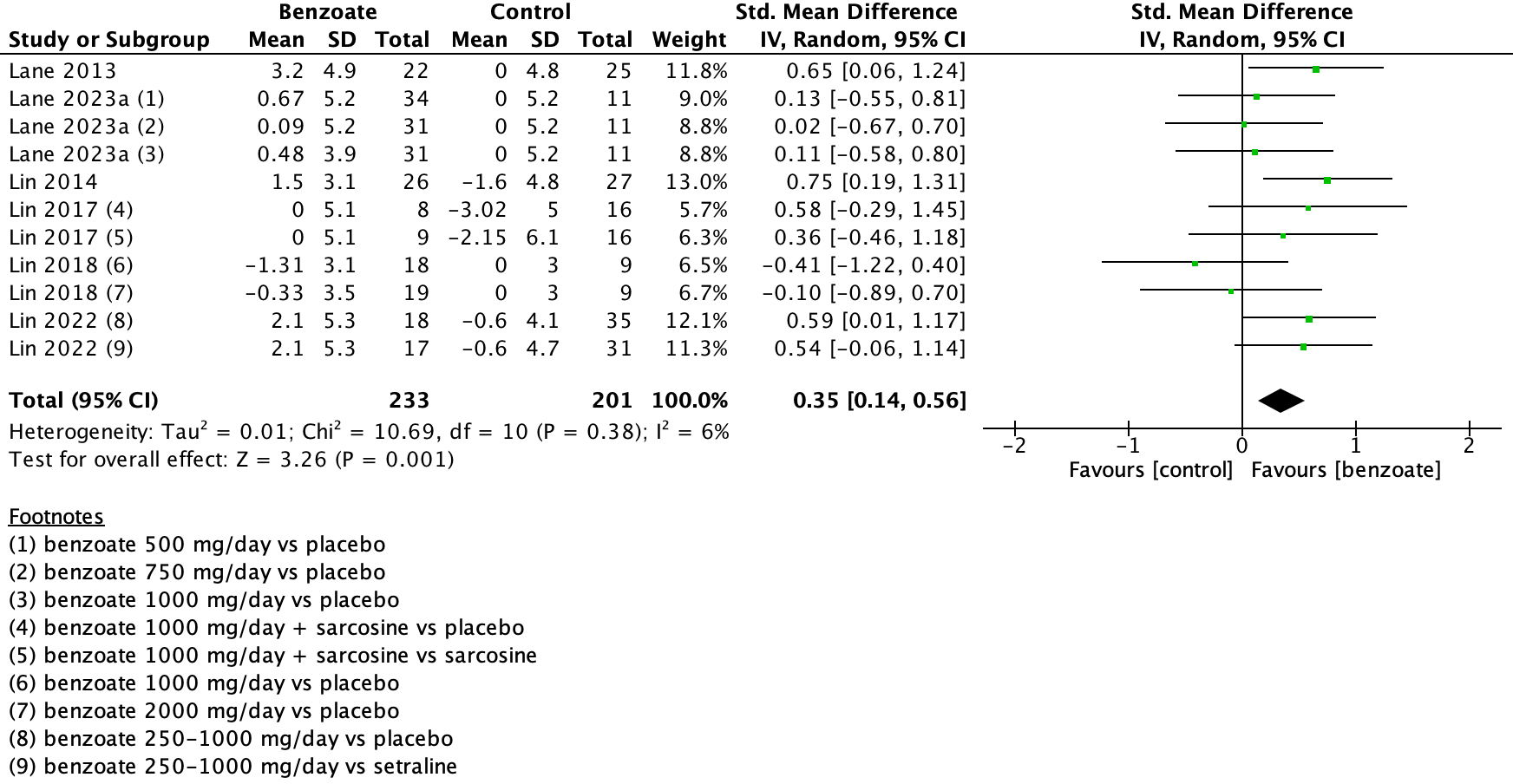
**

1. **Sustained attention**

**
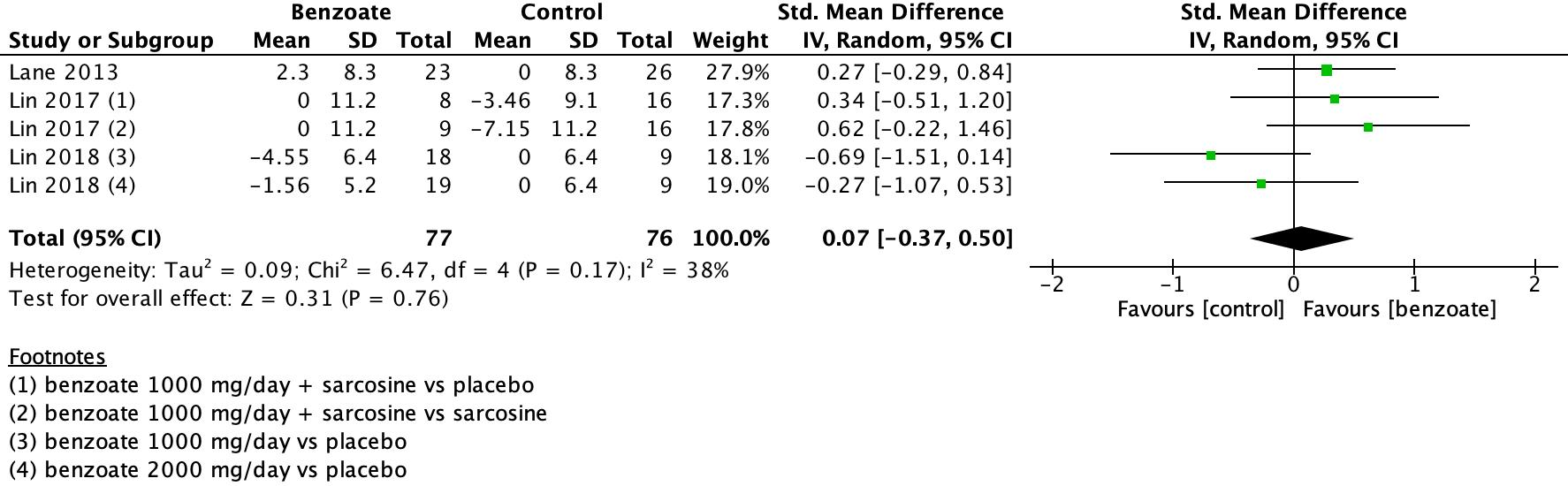
**

1. **Working memory**

**
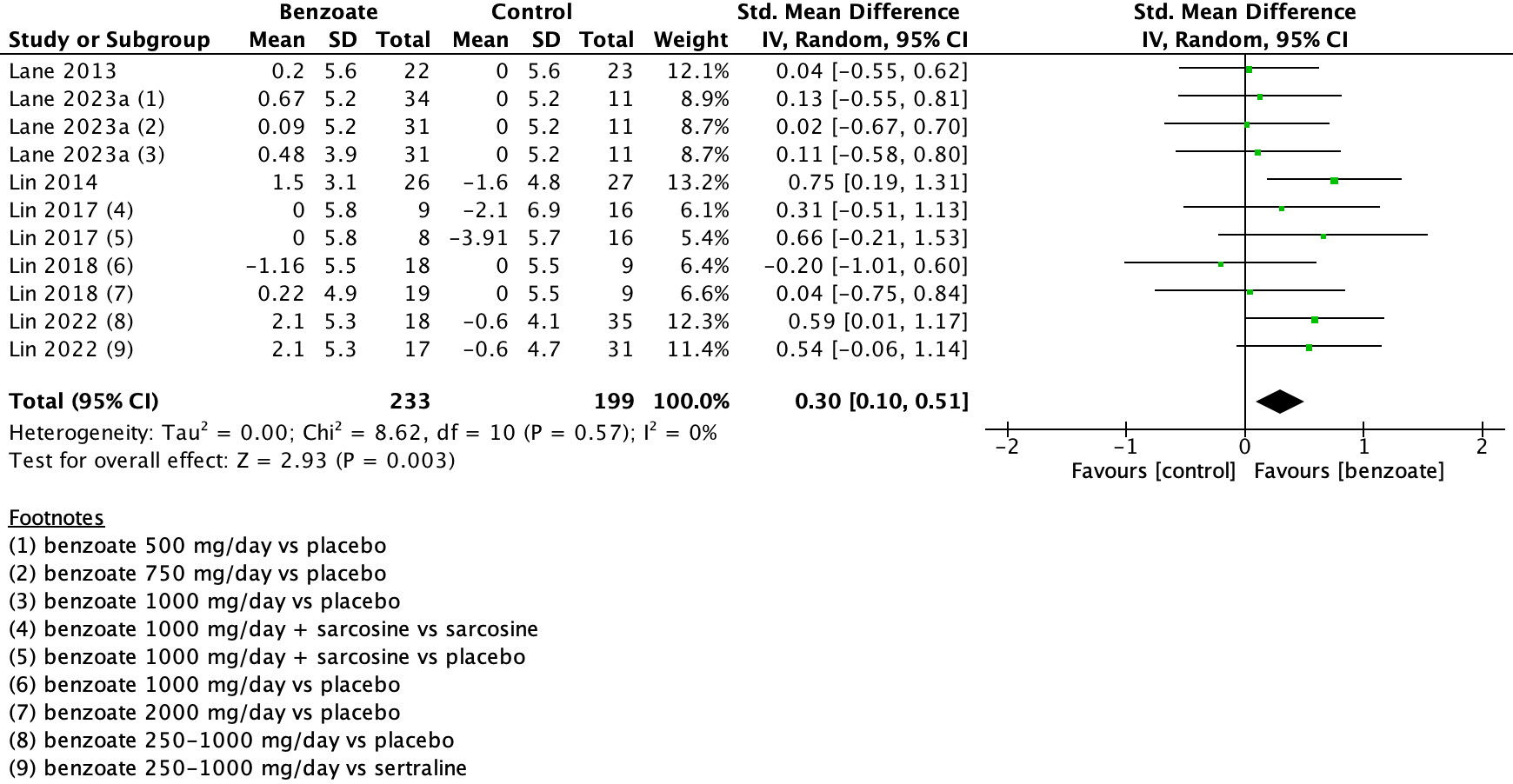
**

1. **Verbal learning and memory**

**
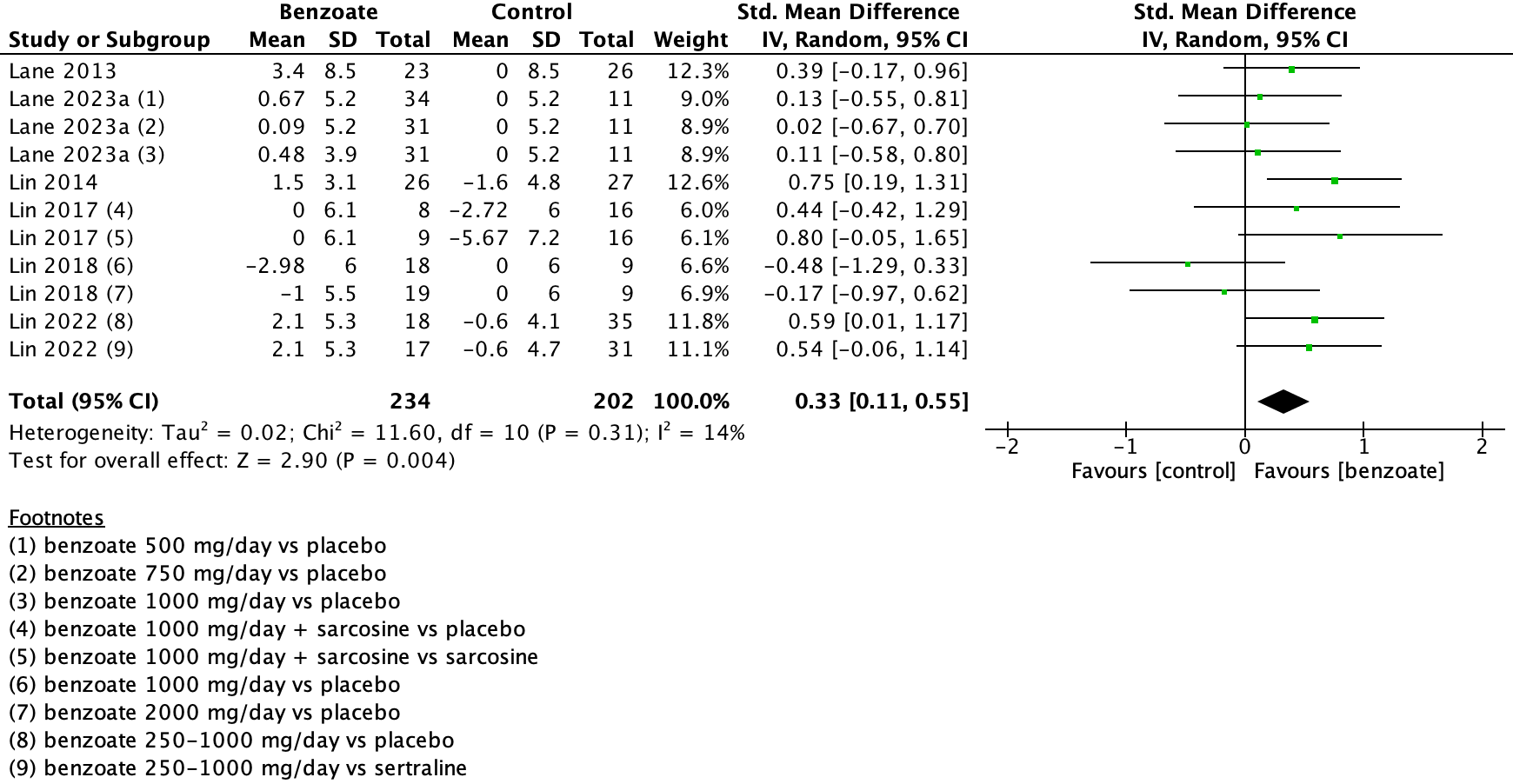
**

1. **Visual learning and memory**

**
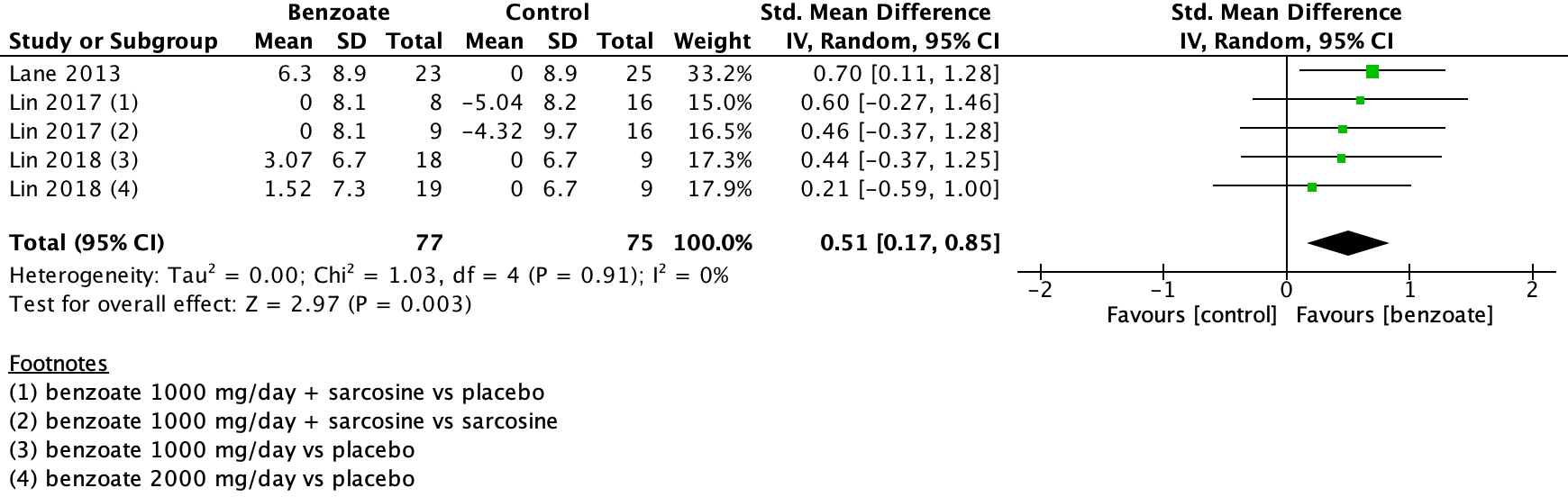
**

1. **Reasoning and problem solving**

**
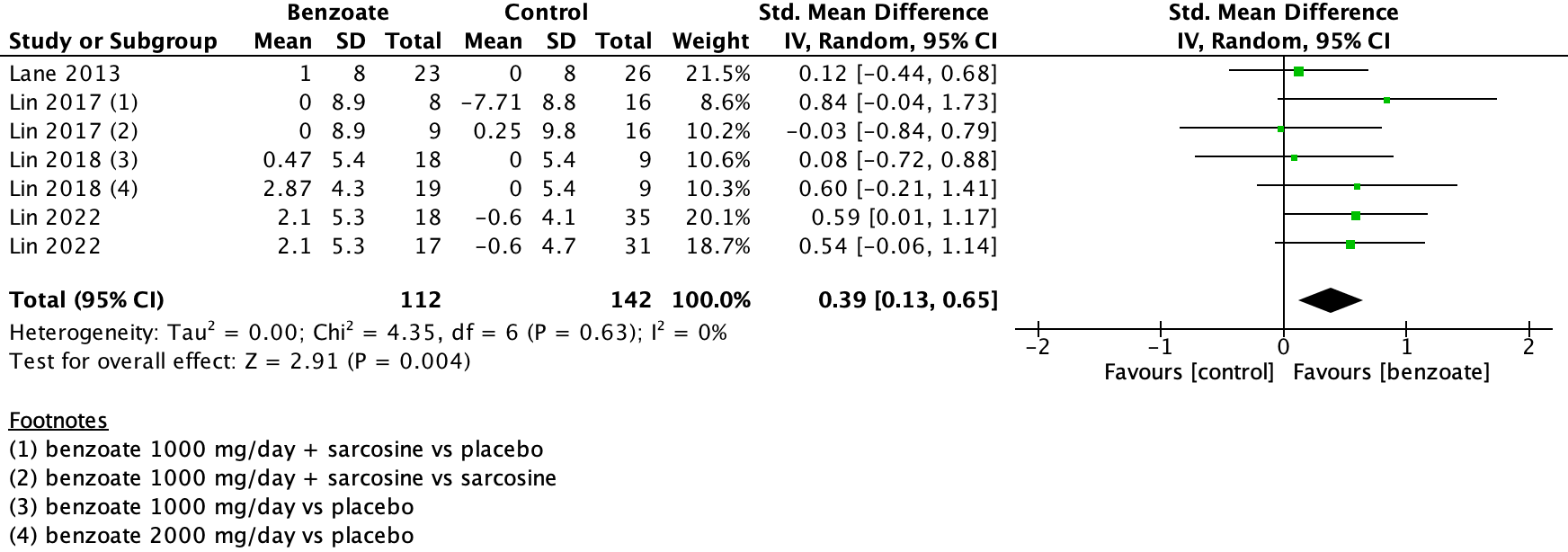
**

1. **Social cognition**

**
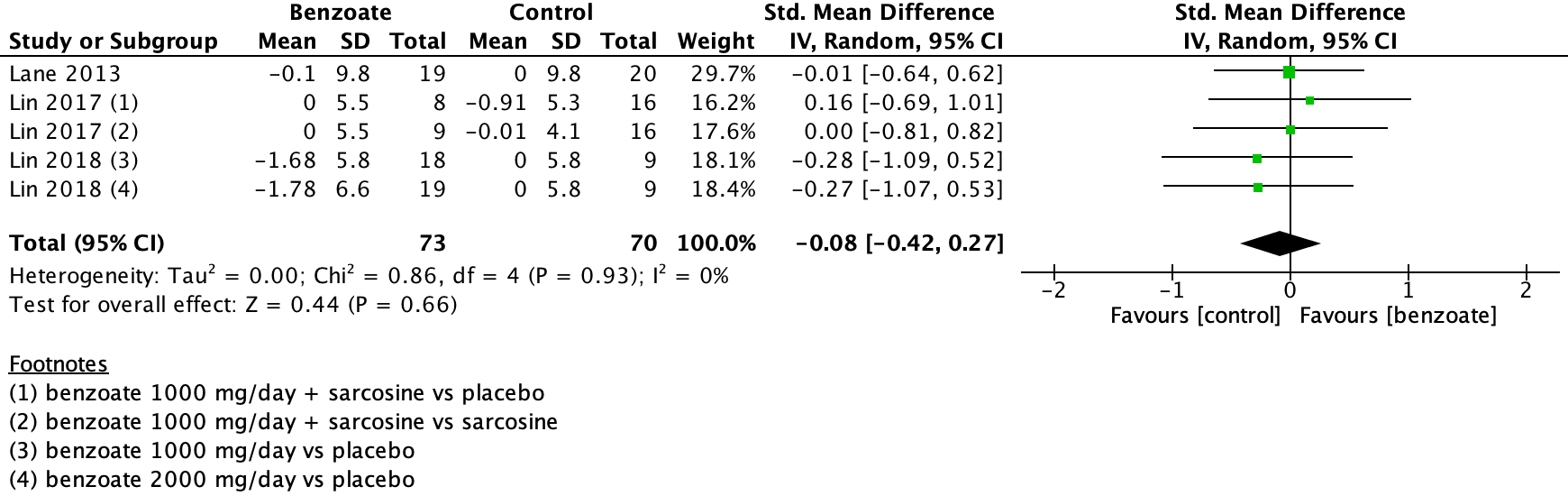
**

The forest plots for included studies pooled together using the random-effects model for assessing difference in (a) speed of procession, (b) sustained attention, (c) working memory, (d) verbal learning and memory, (e) visual learning and memory, (f) reasoning and problem solving, and (g) social cognition. Each point estimate (square) represents the comparison effect (mean difference) of the outcome, and the horizontal lines represent the 95% confidence intervals. Results plotted left of the vertical line indicate effects favoring benzoate. The black diamond represents the combined effect. Abbreviations: CI, confidence interval; df, degrees of freedom; IV, inverse variance; SD, standard deviation.

**Figure S2. Forest plots of the positive and negative symptoms**

1. **PANSS total**


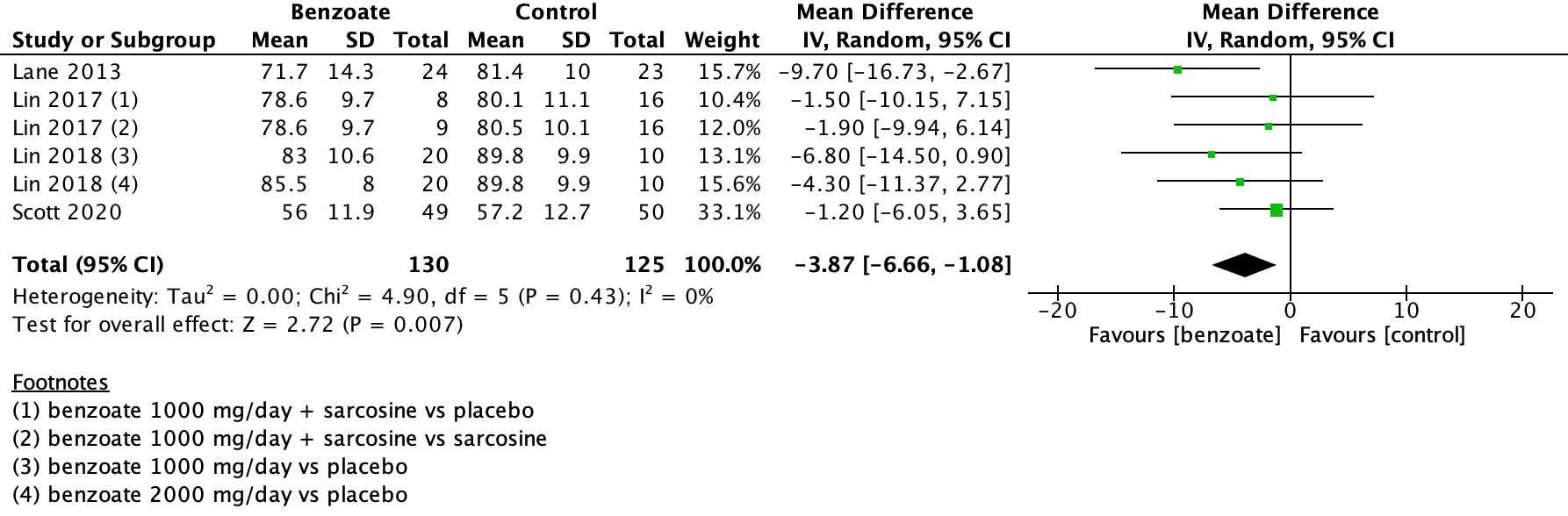


1. **PANSS positive**

**
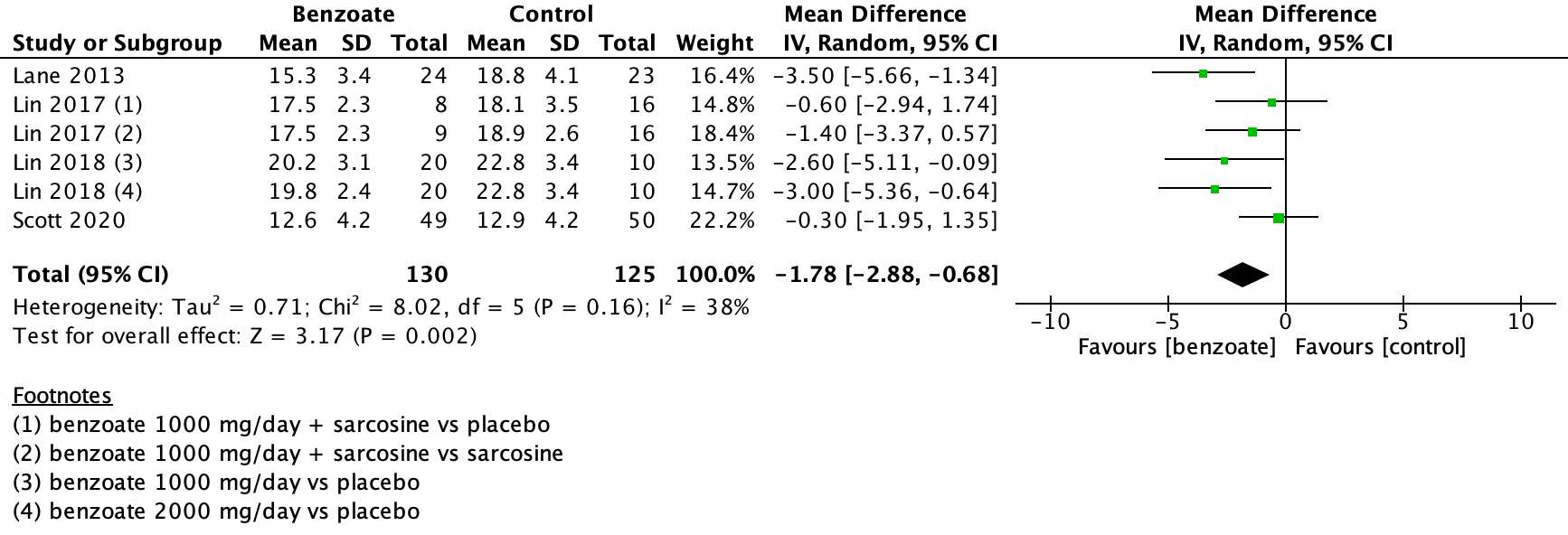
**

1. **PANSS negative**

**
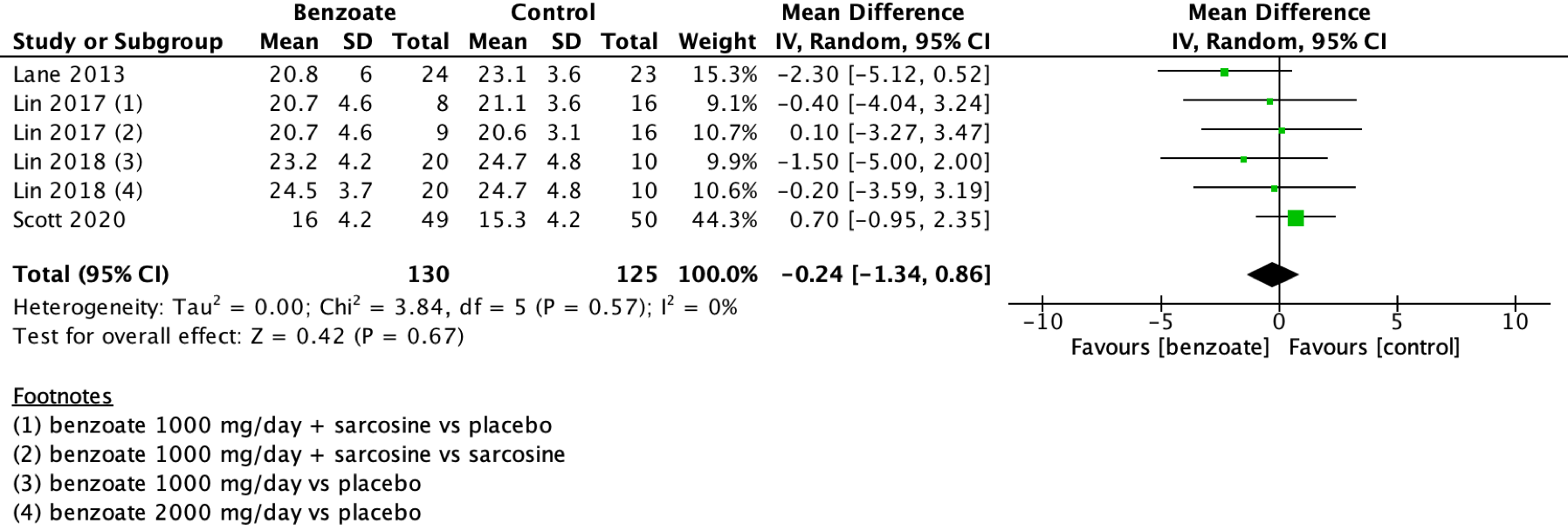
**

1. **PANSS general psychopathology**

**
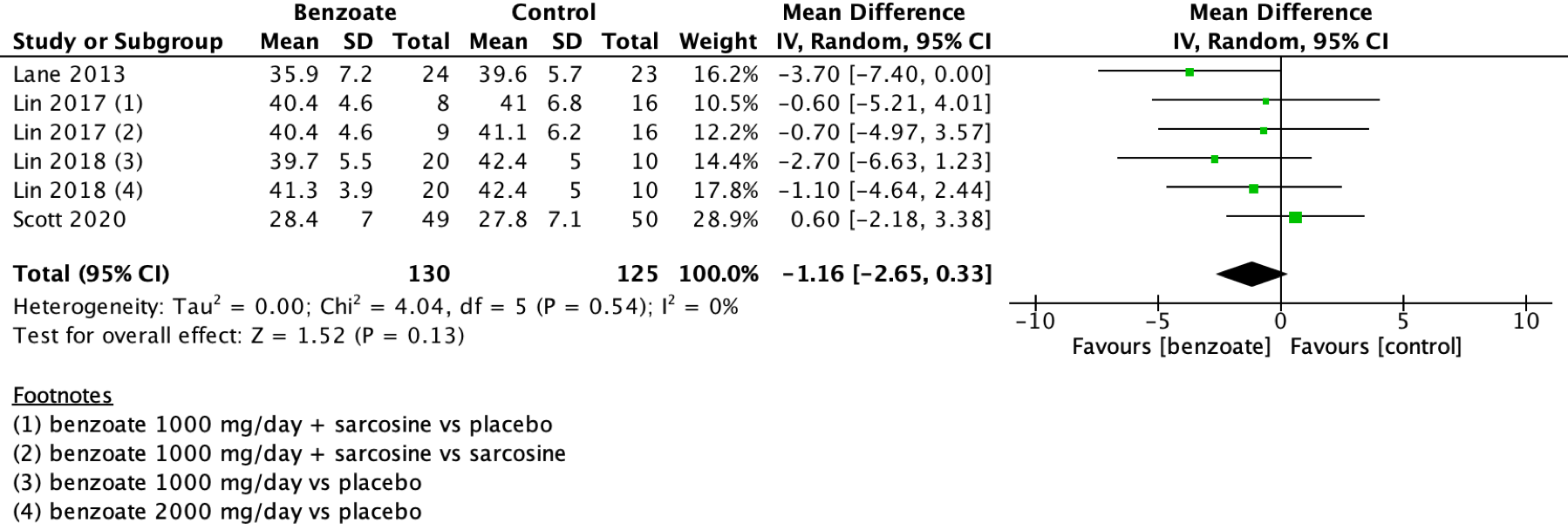
**

The forest plots for included studies pooled together using random-effect model for assessing difference in (a) total PANSS score, (b) PANSS positive scale score, (c) PANSS negative scale score, and (d) PANSS general psychopathology scale score. Each point estimate (square) represents the comparison effect (mean difference) of the outcome, and the horizontal lines represent the 95% confidence intervals. Results plotted left of the vertical line indicate effects favoring benzoate. The black diamond represents the combined effect. Abbreviations: CI, confidence interval; df, degrees of freedom; IV, inverse variance; PANSS, Positive and Negative Symptoms of Schizophrenia Scale; SD, standard deviation.

**Figure S3. Forest plot of depressive symptoms**

**Hamilton Depression Rating Scale**

**
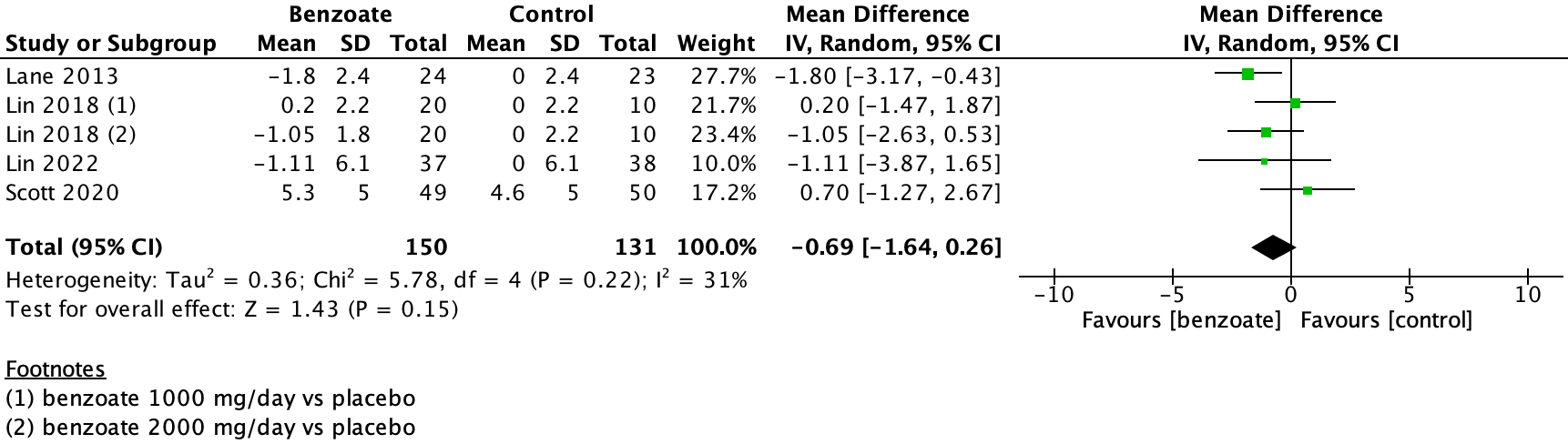
**

The forest plot for included studies pooled together using random-effect model for assessing difference in the Hamilton Depression Rating Scale. Each point estimate (square) represents the comparison effect (mean difference) of the outcome, and the horizontal lines represent the 95% confidence intervals. Results plotted left of the vertical line indicate effects favoring benzoate. The black diamond represents the combined effect. Abbreviations: CI, confidence interval; df, degrees of freedom; IV, inverse variance; SD, standard deviation.

**Figure S4. Forest plots of the safety outcome**

1. **All-cause dropout**

**
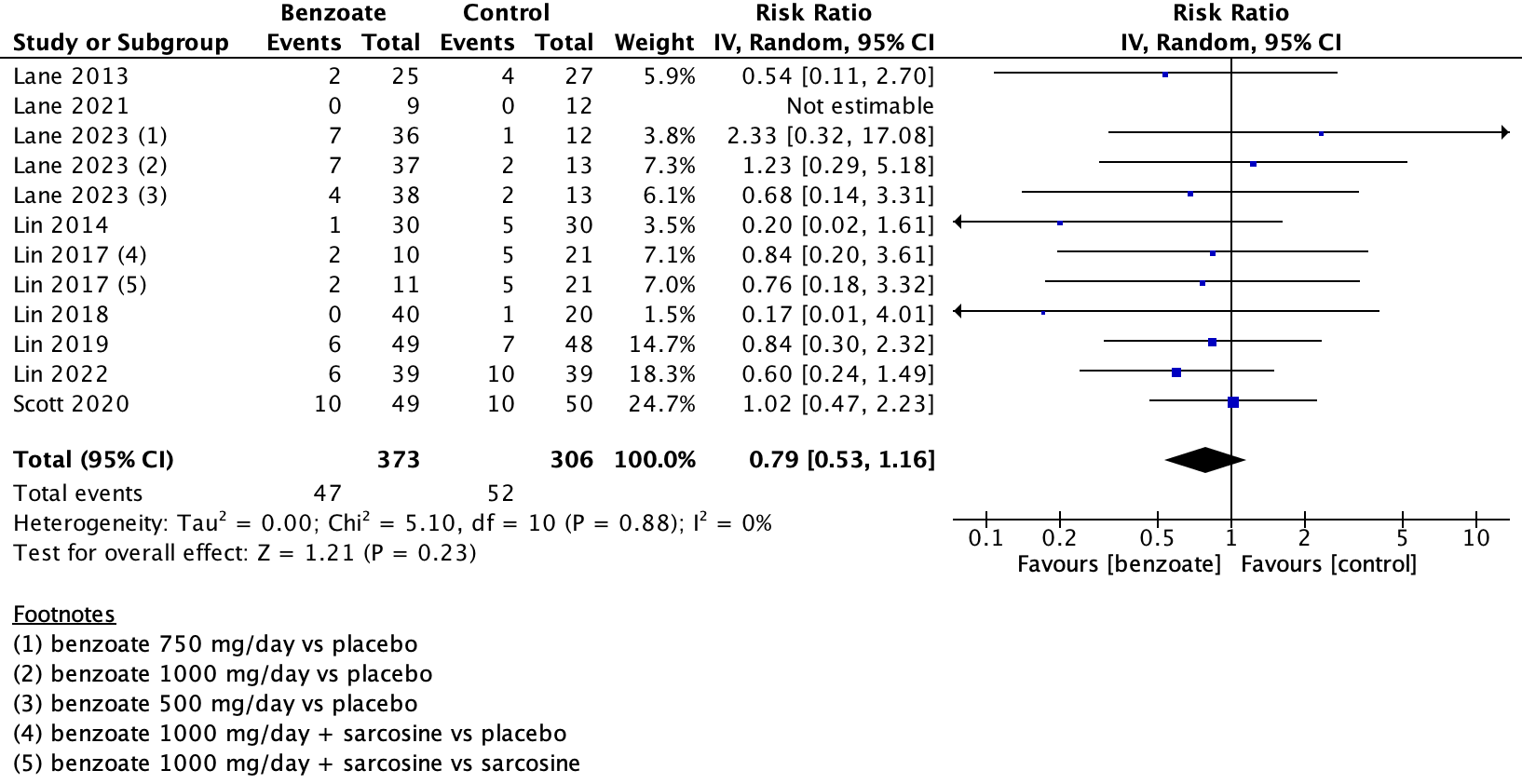
**

1. **All-cause adverse events**

**
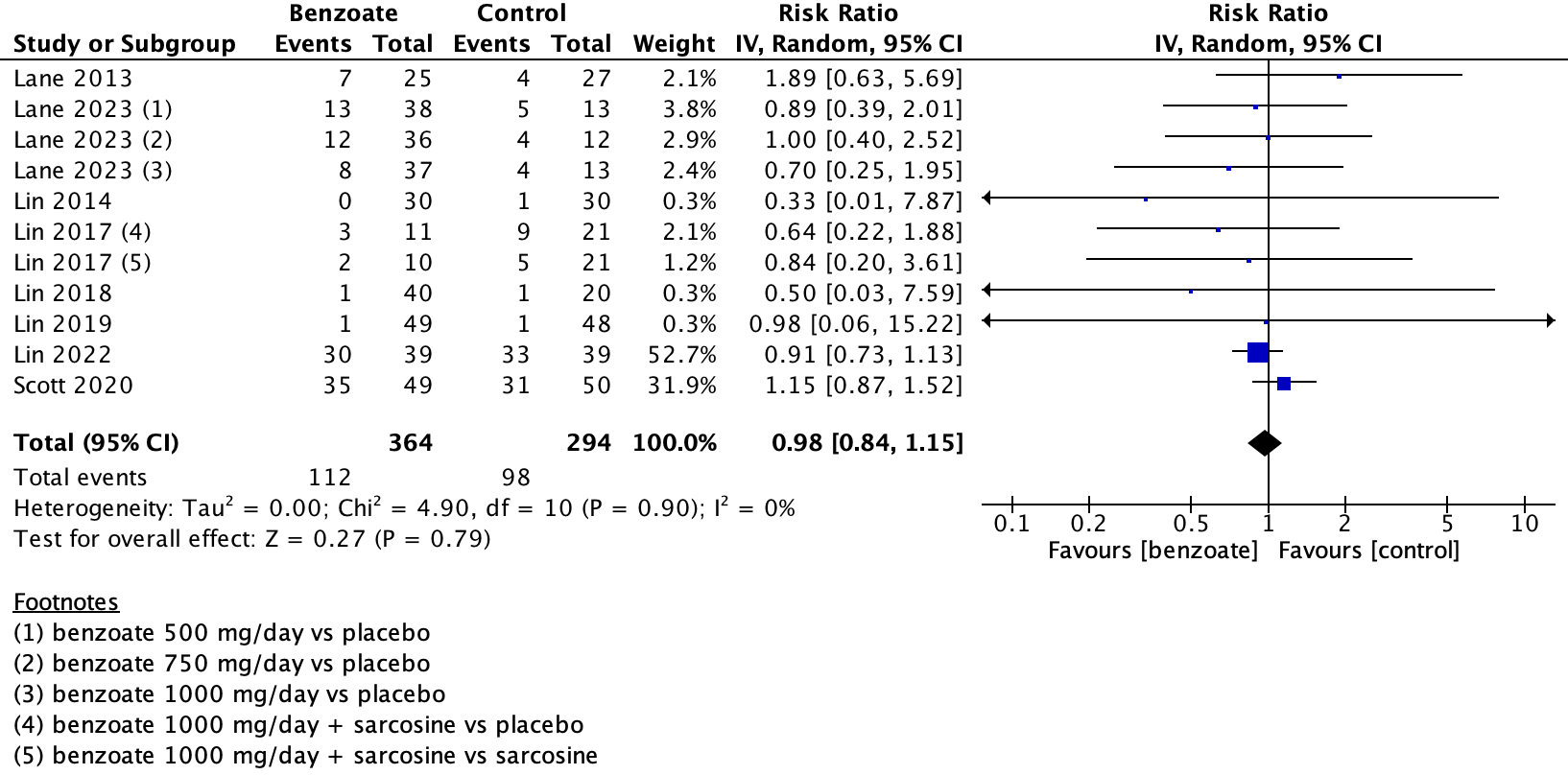
**

1. **Simpson-Angus Scale**

**
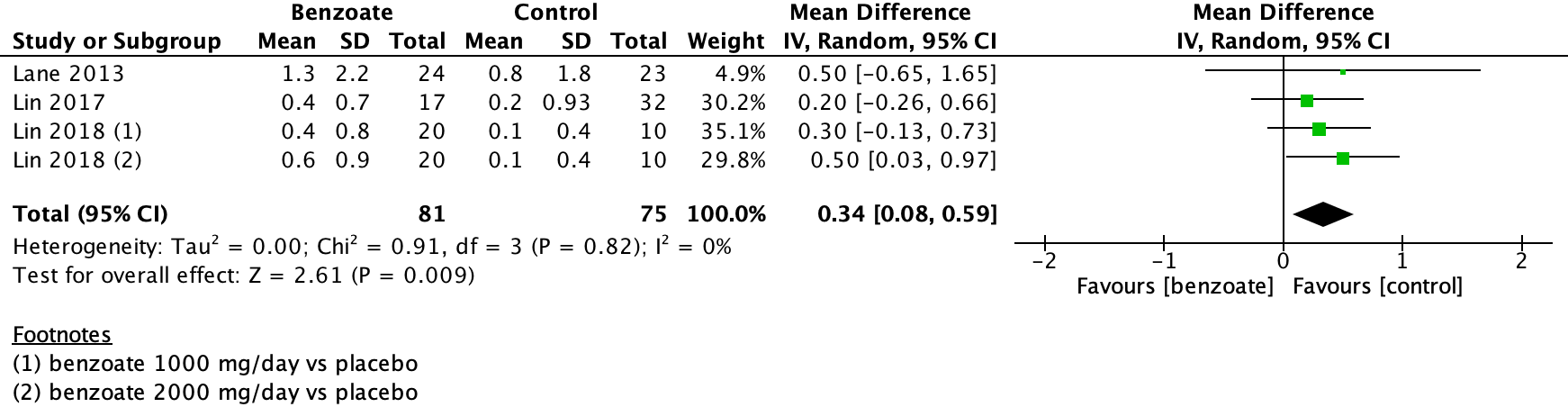
**

1. **Abnormal Involuntary Movement Scale**

**
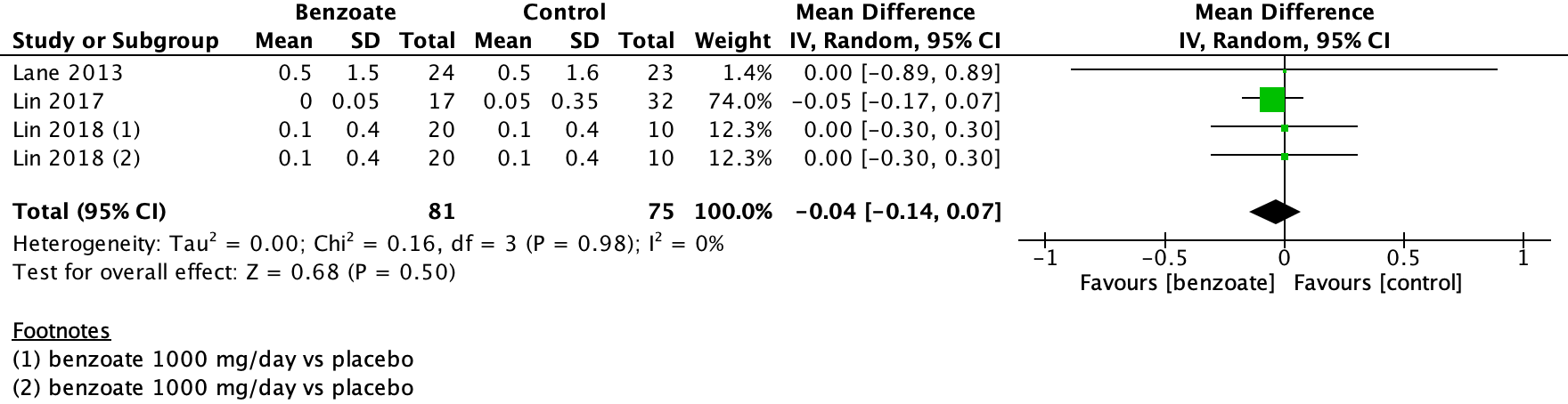
**

1. **Barnes Akathisia Scale**

**
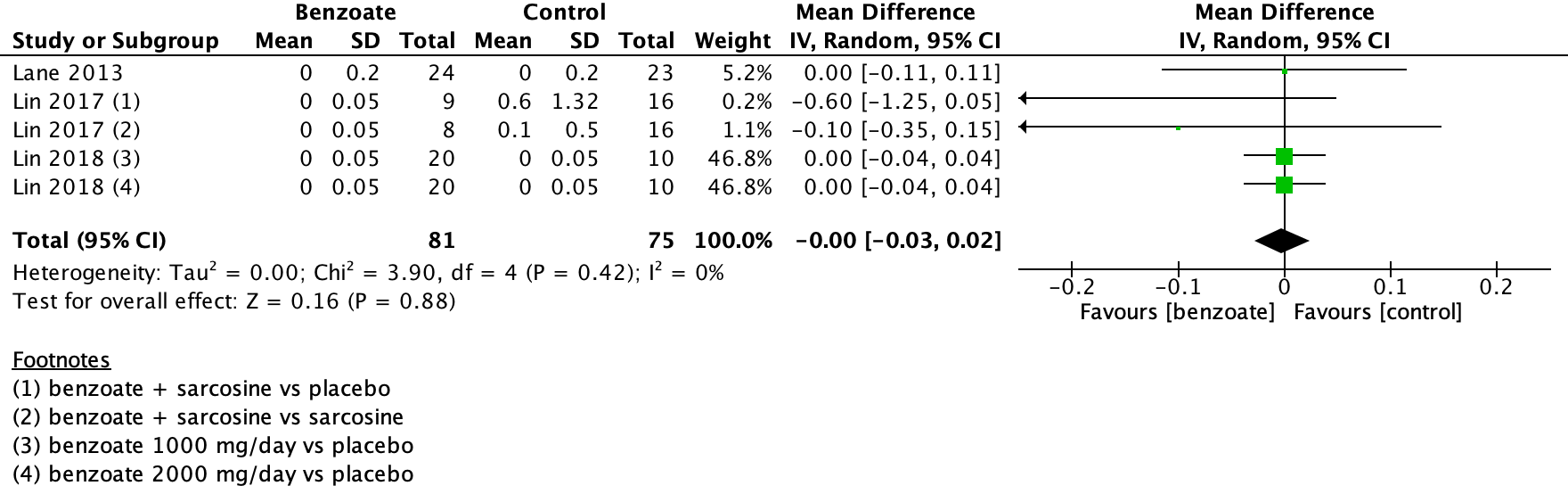
**

The forest plots for included studies pooled together using random-effect model for assessing risk ratio in (a) all-cause dropout and (b) all-cause adverse event and difference in (c) Simpson-Angus Scale, (d) Abnormal Involuntary Movement Scale, and (e) Barnes Akathisia Scale. Each point estimate (square) represents the comparison effect (mean difference) of the outcome, and the horizontal lines represent the 95% confidence intervals. Results plotted left of the vertical line indicate effects favoring benzoate. The black diamond represents the combined effect. Abbreviations: CI, confidence interval; df, degrees of freedom; IV, inverse variance; SD, standard deviation.

**Figure S5. Funnel plots of publication bias**

1. **Global cognitive function**


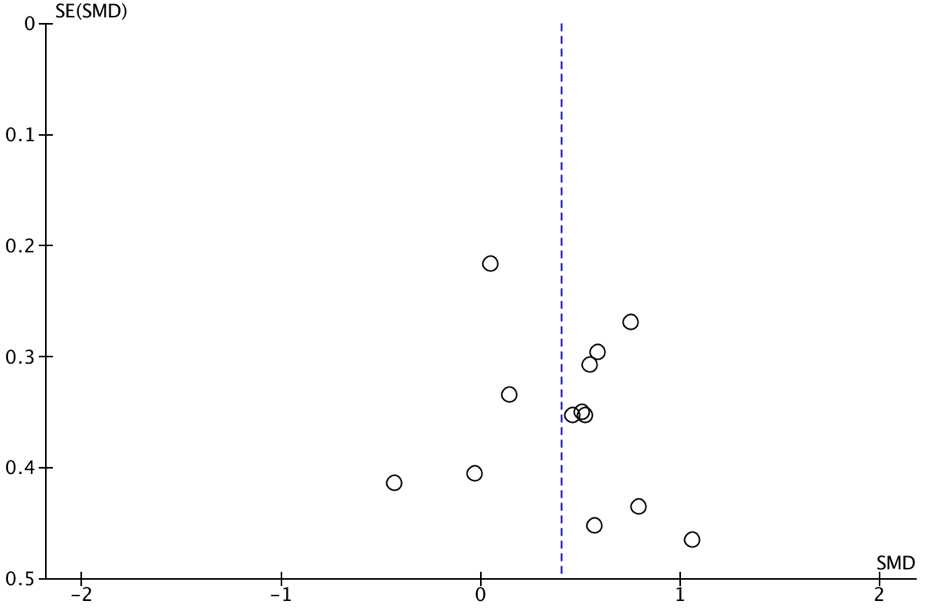


Egger’s test *p* = 0.50

1. **Positive and negative symptoms of schizophrenia**


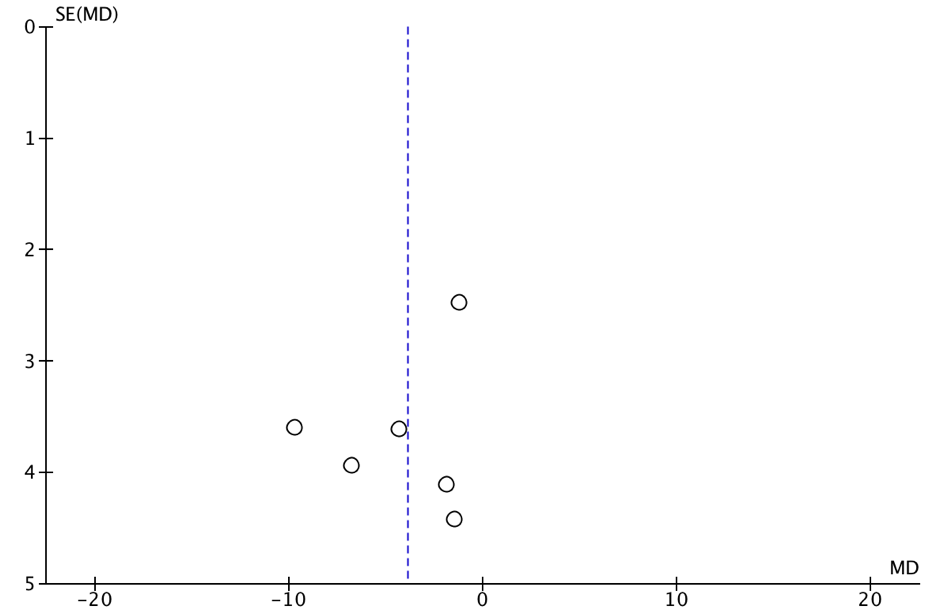


Egger’s test not performed

1. **Hamilton Depression Rating Scale**

**
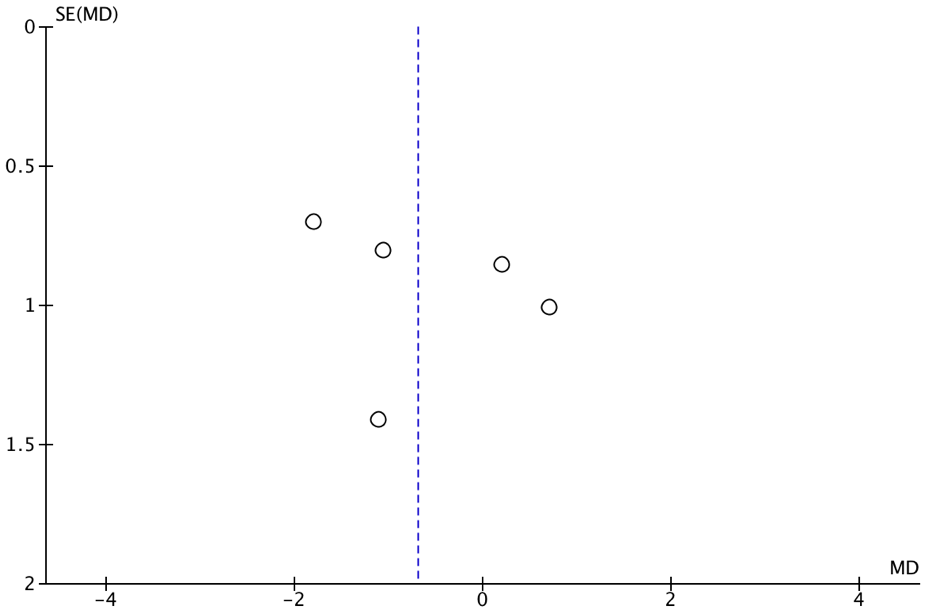
**

Egger’s test not performed

Abbreviations: MD, mean difference; RR, risk ratio; SE, standard error; SMD, standardized mean difference.

**Figure S6. Trial sequential analyses of seven cognitive domains**

1. **Speed of processing**


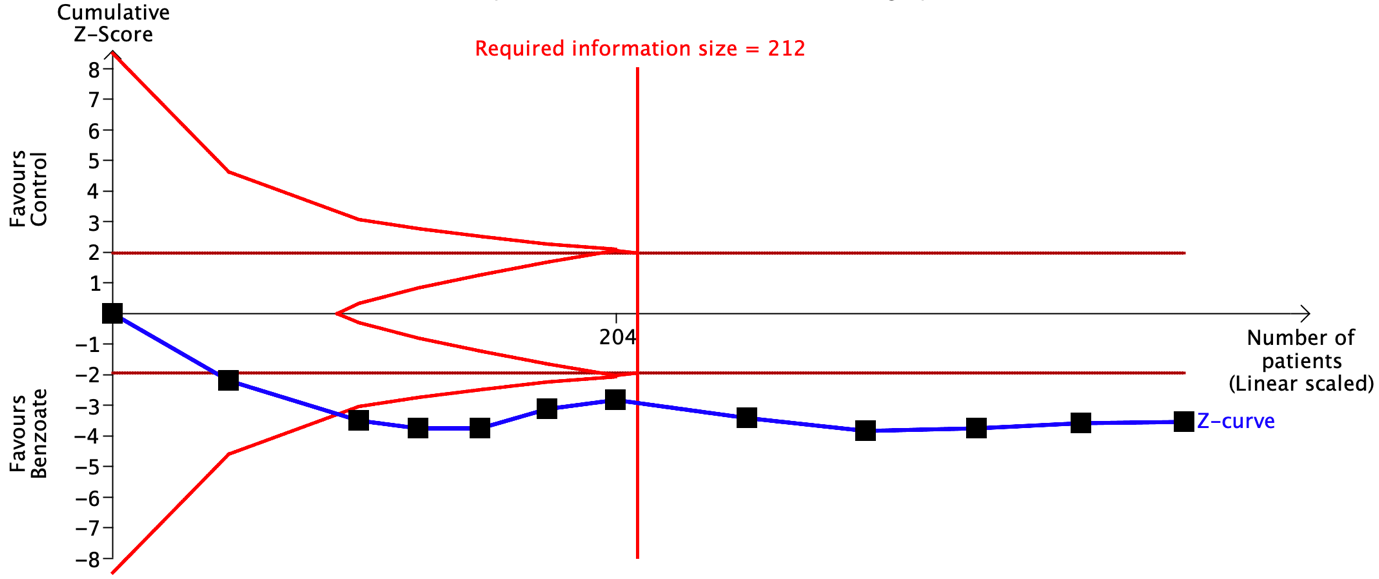


1. **Sustained attention**


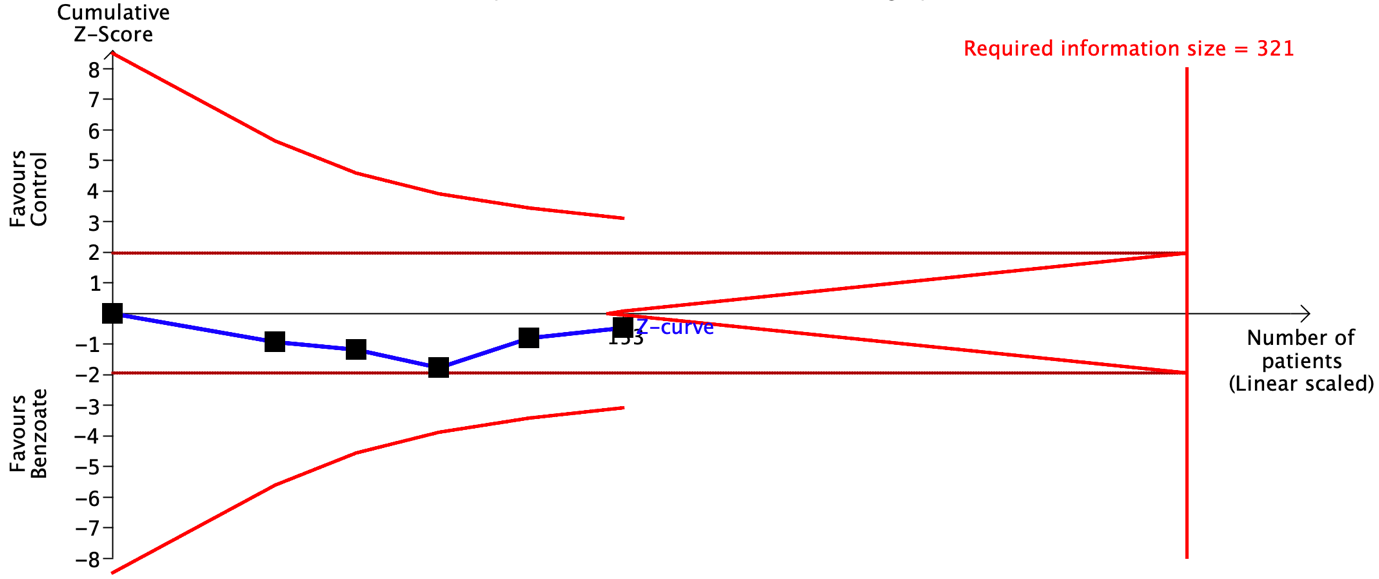


1. **Working memory**


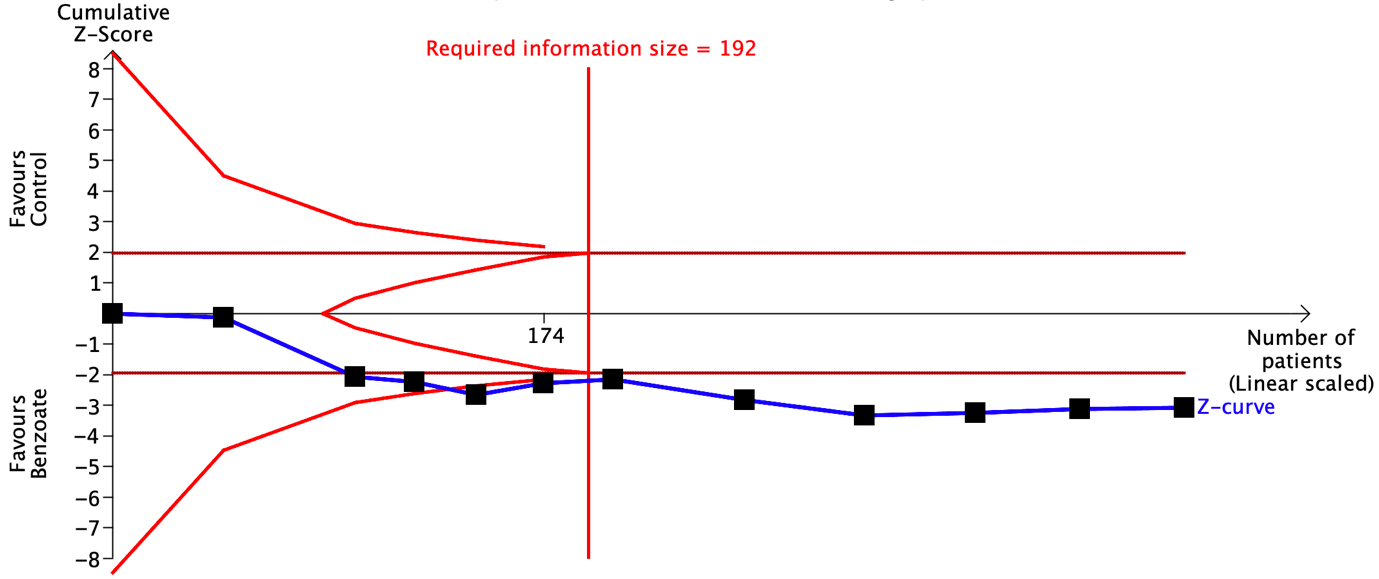


1. **Verbal learning and memory**


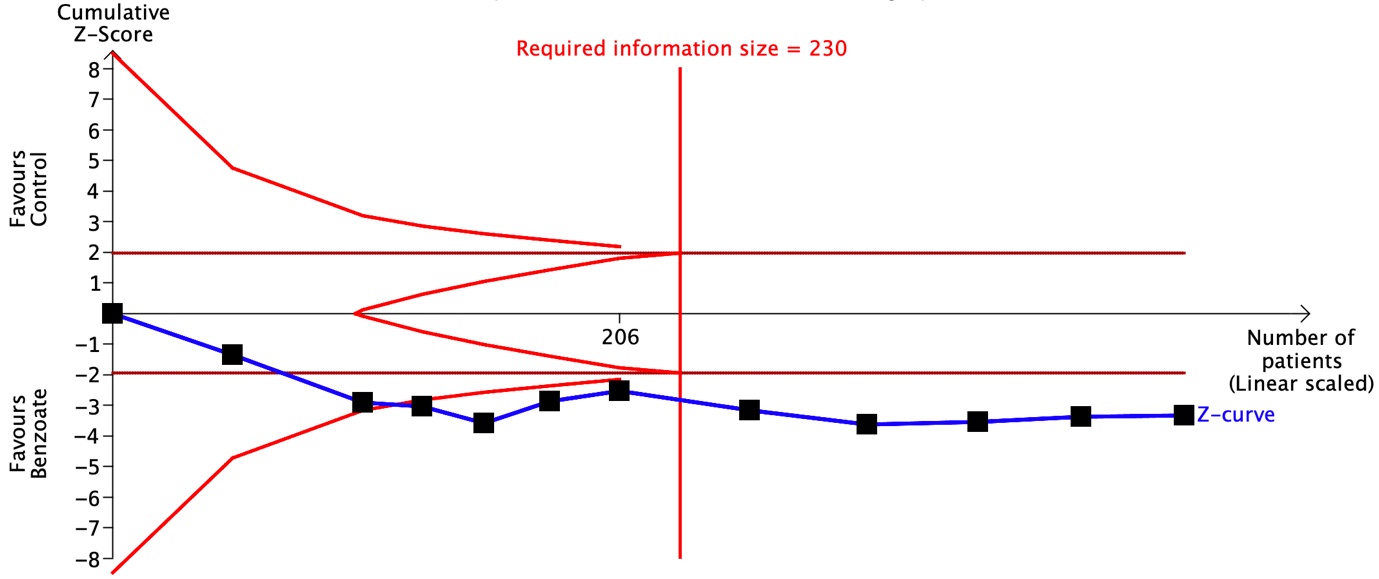


1. **Visual learning and memory**


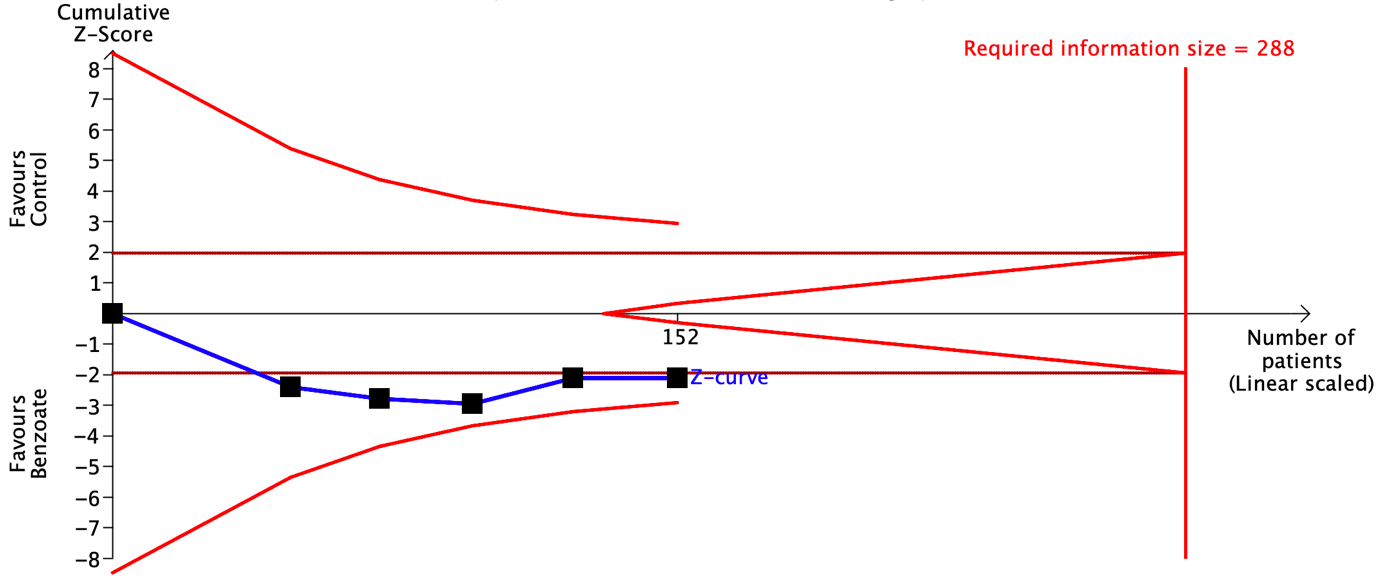


1. **Reasoning and problem solving**

**
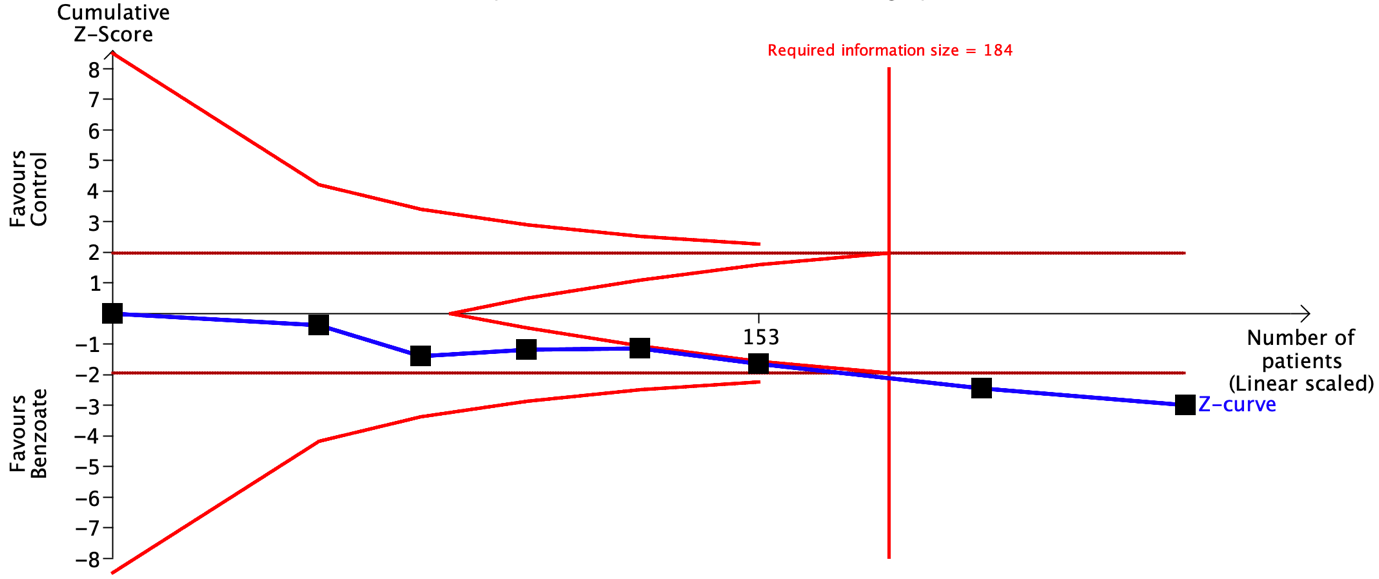
**

1. **Social cognition**

**
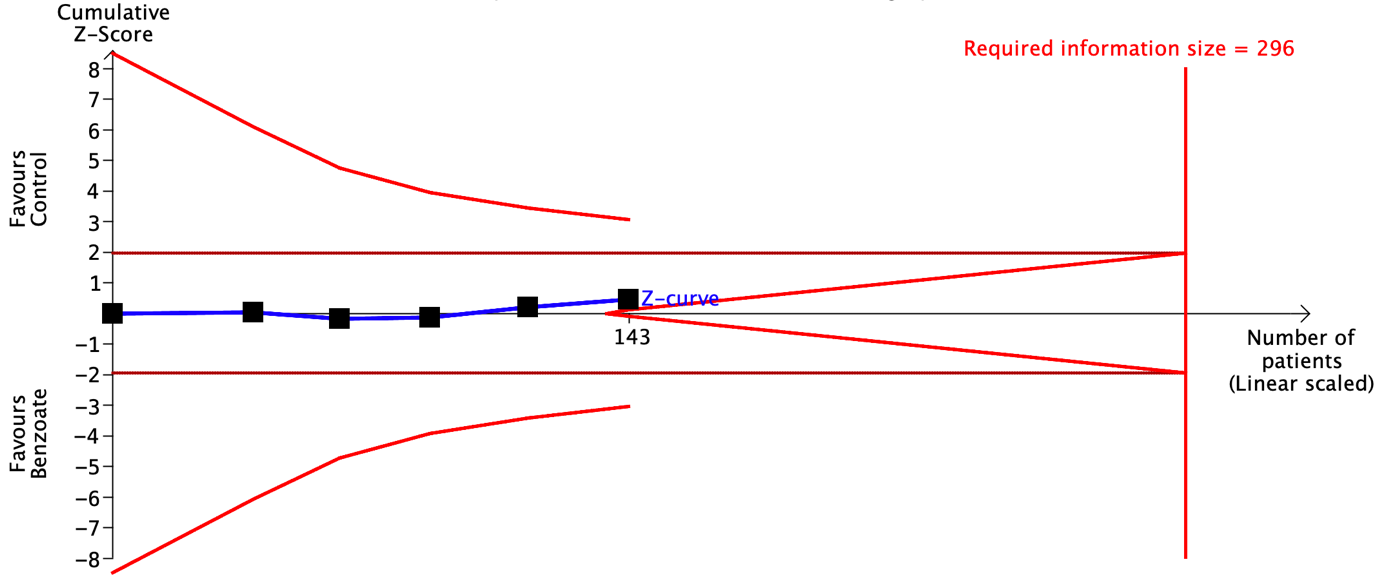
**

With 90% statistical power and 5% type I error, the required information size detects a standardized mean difference of 0.5.

**Figure S7. Trial sequential analyses of secondary outcomes**

1. **Positive and Negative Symptoms of Schizophrenia Scale**

**
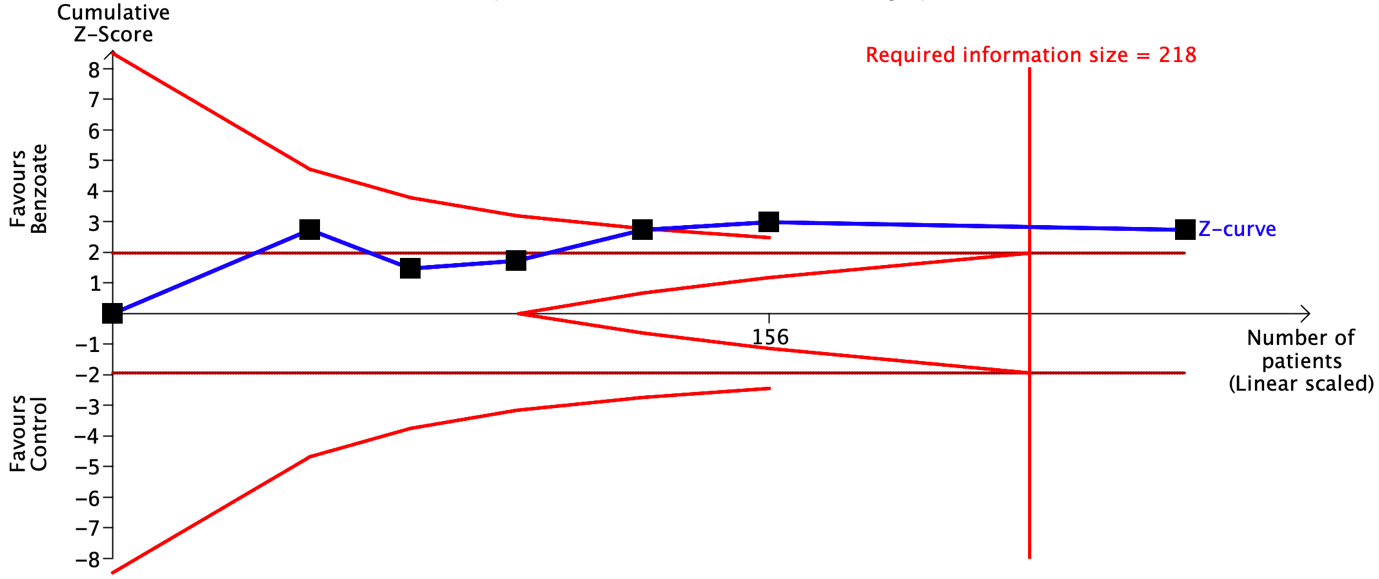
**

1. **Hamilton Depression Rating Scale**

**
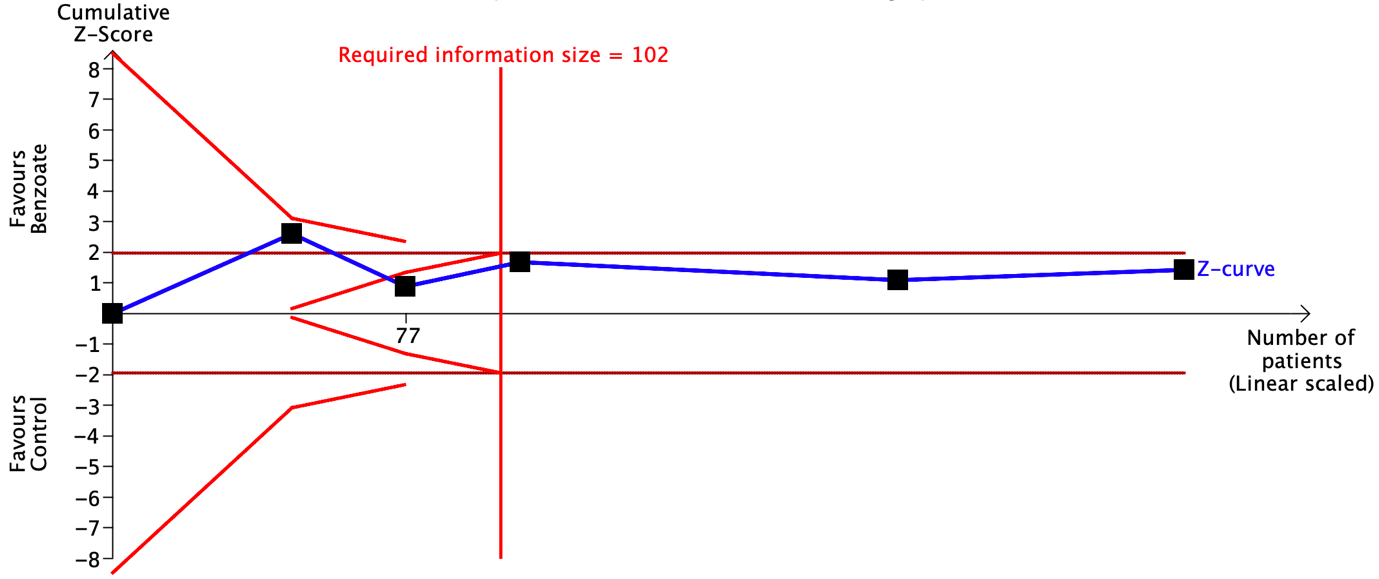
**With 90% statistical power and 5% type I error, the required information size detects mean difference of Positive and Negative Symptoms of Schizophrenia Scale -5 and mean difference of Hamilton Depression Rating Scale -4.
